# Large-Scale Detection of Telomeric Motif Sequences in Genomic Data Using TelFinder

**DOI:** 10.1128/spectrum.03928-22

**Published:** 2023-02-27

**Authors:** Qing Sun, Hao Wang, Shiheng Tao, Xuguang Xi

**Affiliations:** a College of Life Sciences, Northwest A&F University, Yangling, Shaanxi, China; b Bioinformatics Center, Northwest A&F University, Yangling, Shaanxi, China; c State Key Laboratory of Crop Stress Biology in Arid Areas, Northwest A&F University, Yangling, Shaanxi, China; d Université Paris-Saclay, ENS Paris-Saclay, CNRS, LBPA, Gif-sur-Yvette, France; The Ohio State University

**Keywords:** detect telomeric motif sequence, fungi, variation in telomere

## Abstract

Telomeres are regions of tandem repeated sequences at the ends of linear chromosomes that protect against DNA damage and chromosome fusion. Telomeres are associated with senescence and cancers and have attracted the attention of an increasing number of researchers. However, few telomeric motif sequences are known. Given the mounting interest in telomeres, an efficient computational tool for the *de novo* detection of the telomeric motif sequence of new species is needed since experimental-based methods are costly in terms of time and effort. Here, we report the development of TelFinder, an easy-to-use and freely available tool for the *de novo* detection of telomeric motif sequences from genomic data. The vast quantity of readily available genomic data makes it possible to apply this tool to any species of interest, which will undoubtedly inspire studies requiring telomeric repeat information and improve the utilization of these genomic data sets. We have tested TelFinder on telomeric sequences available in the Telomerase Database, and the detection accuracy reaches 90%. In addition, variation analyses in telomere sequences can be performed by TelFinder for the first time. The telomere variation preference of different chromosomes and even at the ends of the chromosome can provide clues regarding the underlying mechanisms of telomeres. Overall, these results shed new light on the divergent evolution of telomeres.

**IMPORTANCE** Telomeres are reported to be highly correlated with the cell cycle and aging. As a result, research on telomere composition and evolution has become more and more urgent. However, using experimental methods to detect telomeric motif sequences is slow and costly. To combat this challenge, we developed TelFinder, a computational tool for the *de novo* detection of the telomere composition only using genomic data. In this study, we showed that a lot of complicated telomeric motifs could be identified by TelFinder only using genomic data. In addition, TelFinder can be used to check variation analyses in telomere sequences, which could lead to a deeper understanding of telomere sequences.

## INTRODUCTION

In contrast to the replication of the prokaryotic circular chromosome, linear chromosome replication in eukaryotes is semidiscontinuous (1), leading to the loss of genetic information per cell replication. Cells have evolved to alleviate this problem through telomeres at chromosome termini. Telomeres help prevent the loss of genetic information at chromosome ends and avoid end-to-end chromosome fusions by distinguishing double-stranded DNA (dsDNA) breaks from the native chromosome ends (2). Most eukaryotes express telomerase, a reverse transcriptase that continuously elongates telomeres (2).

The telomeric repeat sequence represents the smallest unit of the telomere sequence. Most eukaryotes in the same clades carry telomeric repeat sequences conserved in adjacent branches. The telomeric repeat sequence TTAGGG, discovered in Homo sapiens, is common among vertebrates (3). Consensus-type motifs in other groups seem to be variants of the vertebrate-type TTAGGG. For instance, TTTAGGG is found in Arabidopsis thaliana and most land plants (4). Most insects carry the telomeric repeat TTAGG (5, 6). Nevertheless, there are exceptions to the consensus type in some clades. In *Allium cepa* and its related species, a unique motif, CTCGGTTATGGG (7), replaced the Arabidopsis-type and vertebrate-type motifs. In addition, Cestrum elegans lacks the canonical TTTAGGG motif and instead carries an unusual motif, TTTTTTAGGG (8). The evolution and origin of telomeric motifs of different clades have attracted the attention of many researchers (7, 9). However, the limited knowledge of telomeric motif sequences has restricted research into the large-scale evolution and origin of telomeres.

Telomeres are synthesized according to the template sequence on telomerase RNA. Thus, telomeres are composed of the reverse complementary sequence of the template. It is conceivable that telomere composition detection can provide a reference for determining telomerase RNA, further promoting the study of these RNAs (10–12). Telomere length, an indicator of replicative potential, is an essential characteristic of telomeres and is related to human health and disease. Current tools for estimating the length of telomere sequences from whole-genome sequencing (WGS) data require known telomeric repeat sequences (13). In summary, easy and quick detection of telomeric repeat sequences will substantially facilitate these studies.

As mentioned above, identifying telomeric repeat sequences is a cornerstone of many telomere-related fields. However, experimental approaches to detect motifs involve fluorescence *in situ* hybridization (FISH) or Southern blot analyses of terminal restriction fragments (14), which are laborious and time-consuming techniques. Sequence repeat finder (SERF) (15) is a Web-based tool for the *de novo* detection of telomeric repeat sequences. Users can identify telomere content from genomic data. However, there are limitations to the use of SERF. (i) SERF can provide only a list of candidates of kmers ranked by their repeat times rather than the candidates’ reliability. (ii) Uploading input files and running time are affected by network linking. In addition, some other tools perform similar functions. TelomereHunter (16) estimates telomere content and composition from cancer genomes and is limited to vertebrate-type TTAGGG or its variants with single nucleotide mutations.

To address these limitations, we developed TelFinder to identify the telomeric repeat sequences of new species. In addition, variation analyses of telomeric sequences are provided for the first time. TelFinder is suitable for any species, and only genomic data from species with assembled telomeres are required. We have applied TelFinder to all species with known telomeric repeats in the Telomerase Database (http://telomerase.asu.edu). The detection accuracy reaches 90%.

As the telomeric repeat sequences of fungi are divergent and irregular (10), we then applied this algorithm to all 137 fungi with genomic data available in the NCBI genome database (https://www.ncbi.nlm.nih.gov/genome/). Telomeric repeats from 99 fungi were identified successfully, of which 50 had not been reported before. These results support the future analysis of the origin of telomeres and telomerase RNA in fungi. Seemingly irregular telomeric sequences in Saccharomycotina can be variants of TTAGGG. This finding suggests that the ancestor of fungi may carry the telomeric repeat sequence TTAGGG. The telomeric sequence or telomerase RNA of Saccharomycotina species may evolve to other types via unknown mechanisms.

Analyses of telomerase extension products have revealed that the fidelity of telomere replication is not absolute (17, 18). For example, T deletion is the most common variation type in the telomere sequence in green algae (19). Our investigation of telomere sequences also revealed mutations and indels, which may correspond to the telomerase error rate. These variations might be related to phylogeny positions and cell states. Therefore, effective methods are needed to detect variations in the telomere region. Users can quickly identify telomere variation from existing genomic data using TelFinder, including variation frequency and preference, avoiding the time and effort required for experiment-based tools.

Overall, TelFinder is a freely available tool for the *de novo* detection of the telomeric repeat sequence of any species with available genomic data, which is beneficial for telomere research. Determining the basic statistics of variation in telomere sequences will also provide insights to researchers studying telomere biology.

## RESULTS

### Overview of the detection principle of TelFinder.

TelFinder is developed for the *de novo* detection of the base composition of telomeric motif sequences using genomic data, which can help users to get telomere content information of any species they are interested in. The number of repeats at the chromosomal termini is an important basis for the detection.

**FIG 1:**
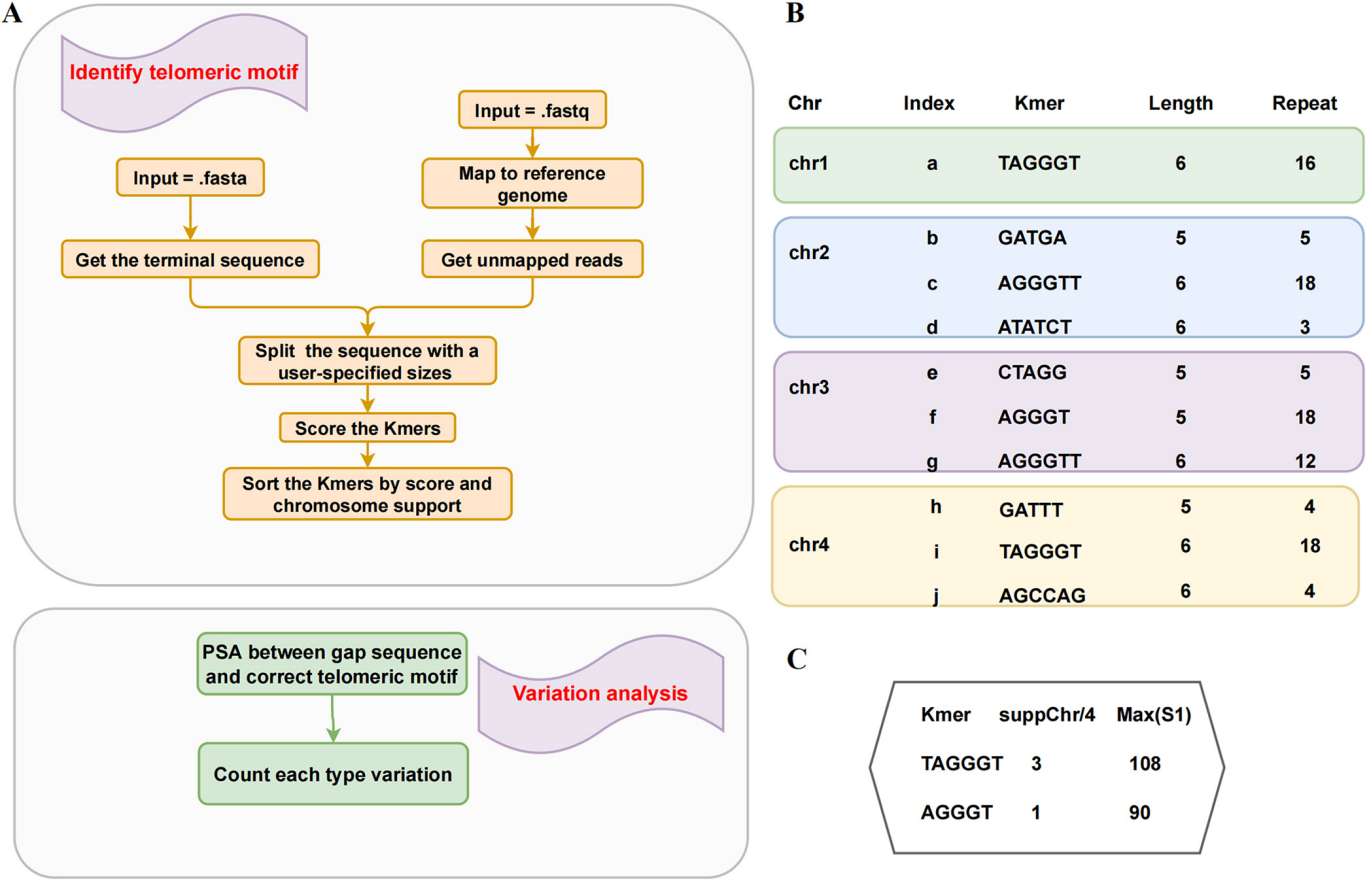
Overview of the basic principle of TelFinder. (A) Schematic diagram of the telomeric motif sequence identification process in TelFinder. (B) A toy example of scoring. The kmer length and the repeat counts are listed for each chromosome. (C) The ranking of the kmer candidates. Two scores, namely, max(Value) and suppChr, were calculated to rank the kmers. The detailed calculation is described in the Materials and Methods section. Max(Value) represents the maximum Value among all chromosomes.

The overview of the principle of TelFinder is shown in Fig. 1. The input file can be either assembled genomes (Fasta format) or raw read data (Fastq format). The telomere information should be included in the assembled genomes or raw reads for successful detection. Then the sequences of a length specified by the user are cut as the search range. As the telomere is hard to assemble for a set of genome sequencing methods, it is recommended to detect telomere content using raw read data when too short telomere sequences are assembled. The telomeric reads can be among unmapped reads as short telomere sequences are included in the reference genome. Therefore, the search is performed in unmapped reads in the Fastq option. Then the sequences or the reads are split into short kmers of the user-specified length range. The most important aspect is to score these kmers, as shown in Fig. 1B. The scoring principle considers mainly the kmer length and its repeat times. In addition, when two kmers are compared, if one kmer is a substring of the other, the substring kmer needs to be given a corresponding penalty (see Materials and Methods section for details). Another important determinant is the number of chromosomes that recommend the kmers, which is called suppChr. The kmers with the highest suppChr value will be considered the most likely telomeric motif sequence.

### Applying TelFinder to a reference data set.

The telomeric repeat sequences reported in the Telomerase Database (http://telomerase.asu.edu) were used as the reference data set to verify the performance of TelFinder. As mentioned above, the most likely telomeric motif sequence was identified using TelFinder by searching the most frequently repeated sequences at the ends of chromosomes. Thus, in theory, the telomere content can be identified successfully if the analyzed data contain telomere sequence information.

Among the 134 species (those without specific species information were excluded) with telomere composition information reported in the Telomerase Database, 43 had an assembly level higher than the “chromosome” and were tested by TelFinder, which requires this feature. The telomeric motif sequences of 36 species were successfully identified by TelFinder, indicating that these genomes have detectable telomeres. Thirty-two out of the 36 species were consistent with the database records (Table 1). However, this information does not fully represent the accuracy of TelFinder, as additional support was obtained in subsequent studies. The detection results of three species were inconsistent with the Telomerase Database data; however, these results are consistent with subsequent findings. The telomeric motif sequence of Yarrowia lipolytica is TTAGTCAGGG. In contrast, ACGATTGGG is recorded in the Telomerase Database. However, experimental validation (20) supported our finding. TelFinder showed that the telomeric motif sequence of Theileria annulata is TTTAGGG rather than TTTTAGGG, as recorded in the Telomerase Database. However, it has been reported that both can be present (21). Giardia lamblia carries TAGGG (22) at its chromosome termini rather than TTAGG, as recorded in the Telomerase Database. The corresponding references in the Telomerase Database were checked to explore the reasons for the differences between the two findings. The database record for *T. annulata* incorrectly refers to the experimental identification of *T. parva*. No telomere information on G. lamblia was found in the reference cited in the database. Regarding Y. lipolytica, the telomere content recorded in the database was determined by directly checking the terminal sequences of chromosomes. However, rechecking the genome assembly used in that study did not reveal any consecutive motifs at both chromosomal ends. The experimental data supporting the results obtained using TelFinder indicate that the method is reliable. Detailed detection results are shown in Table S1 in the supplemental material.

**TABLE 1:** Detection of telomeric motif sequences in the reference data set

Taxonomy group	No. of species:			
	In DB	With detectable telomeres in their genome	Tested by TelFinder	With accordance
Fungi	38	26	20	18
Invertebrates	66	6	6	6
Plants	8	2	2	2
Protists	8	8	8	6
Amoeba	3	1	0	0
Ciliates	11	0	0	0
Total	134	43	36	32

### *De novo* detection of telomeric repeats in fungi.

Fungi have more complicated telomere sequences than other eukaryotes. Most of these sequences are long and seemingly irregular. However, few fungi have telomere information recorded in the Telomerase Database or experimentally validated, limiting research in this area. Since only genomic data with assembled telomeres are required, it is possible to use TelFinder to study the evolution of fungal telomere sequences. Telomeric motif sequences of 99 out of 134 fungi were successfully identified (see Table S2 in the supplemental material). Among them, 80 had not been reported previously.

Consistent with previous studies, most species in Pezizomycotina, the largest subphylum within Ascomycota, carry the vertebrate-type telomere TTAGGG. Species in the genus Aspergillus have distinct telomeric motif sequences. Aspergillus fumigatus and Aspergillus nidulans have TTAGGG. In contrast, the vertebrate-type telomere is substituted by an unusual motif, TTAGGGTCAACA, in Aspergillus sojae, Aspergillus parasiticus, Aspergillus oryzae, and Aspergillus flavus. In addition, we also found three unreported exceptions within Pezizomycotina. In Peltaster fructicola, the less T-rich telomeric motif sequence TAGGG is present. Another fungus, Drechmeria coniospora, was found to carry CCGTTGCTGTTG rather than TTAGGG. The *Arabidopsis*-type motif TTTAGGG was detected in Penicillium polonicum.

In contrast to Pezizomycotina, telomere content sequences in Saccharomycotina are more divergent than any clades of eukaryotes. Species within the genus *Saccharomyces* have variable-length telomeric repeat sequences T(G)_2-3_(TG)_1-6_ (10). In addition, novel telomeric motif sequences in 53 budding yeast species were determined in this study. These motifs are much longer than those in Pezizomycotina and are often irregular, as shown in Table S2. The telomeric motif sequences in this branch diverge to the extent that even species in the same genus carry distinct motifs. For instance, *Metschnikowia* aff. *pulcherrima* carried telomeres comprising TTAGGGAGGTACGGGTGTCTTAGCATC, and TTAGGGATGTACTGATTTATC was detected in Metschnikowia reukaufii. Distinct telomeric repeat sequences were also observed in the genus *Candida*. TAAGGATGTCACGATCATTGGTG was detected in Candida tropicalis and Candida albicans, while TACGGATGTCTAACTTCTGGTG was detected in Candida dubliniensis. This finding suggests that the telomeric repeats of fungi can vary within one genus.

The telomeric motif sequences of 21 fungi in Basidiomycota were also determined by TelFinder, of which 18 have never been reported. Most of these species have the vertebrate-type motif TTAGGG. Some carry variants of TTAGGG. For example, TTAGGA was found in Malassezia furfur. TTGGA comprises the telomeres of Hericium erinaceus. TTAGTG was detected in Malassezia restricta and Malassezia globosa. In Agaricus bisporus, TTAGGGGG was considered the potential telomeric repeat sequence.

### Divergent telomeric repeat sequences in Saccharomycotina.

The telomeric motif sequences of 54 species in Saccharomycotina were detected successfully. The phylogenetic relationship of these fungi in Saccharomycotina was constructed using single-copy core genes to study the evolution of their telomeres.

In most cases, yeasts in the same genus share consensus telomeric repeat sequences, similar to land plants and animals (10–12). Fungi in the genus *Saccharomyces* carry the motif T(G)_2-3_(TG)_1-6_. Eremothecium gossypii has a unique 24-nucleotide (nt) telomeric repeat sequence, TCTCTCAGCGGTGTGGTGTACGGG. This motif was also identified in another species in the genus *Eremothecium*, namely, Eremothecium cymbalariae. The genetic distance among *Eremothecium* species was considerable, indicating that the motif was conserved in this clade. *Saccharomycetaceae* sp. “*Ashbya aceri*” is known to belong to the family *Saccharomycetaceae*, but the specific genus is unknown. It can be inferred that it belongs to the genus *Eremothecium* by genetic distance. The telomeric repeat sequence of *Saccharomycetaceae* sp. is the same as that of *E. cymbalariae*, indicating that it belongs to the genus *Eremothecium*. The fungi in the genus *Kluyveromyces*, Kluyveromyces lactis, and Kluyveromyces marxianus share an unusual 25-nt motif, ACGGATTTGATTAGGTATGTGGTGT. The yeasts in the genus *Candida*, Candida tropicalis, and Candida albicans carry the motif GGATGTCACGATCATTGGTGTAA.

**FIG 2:**
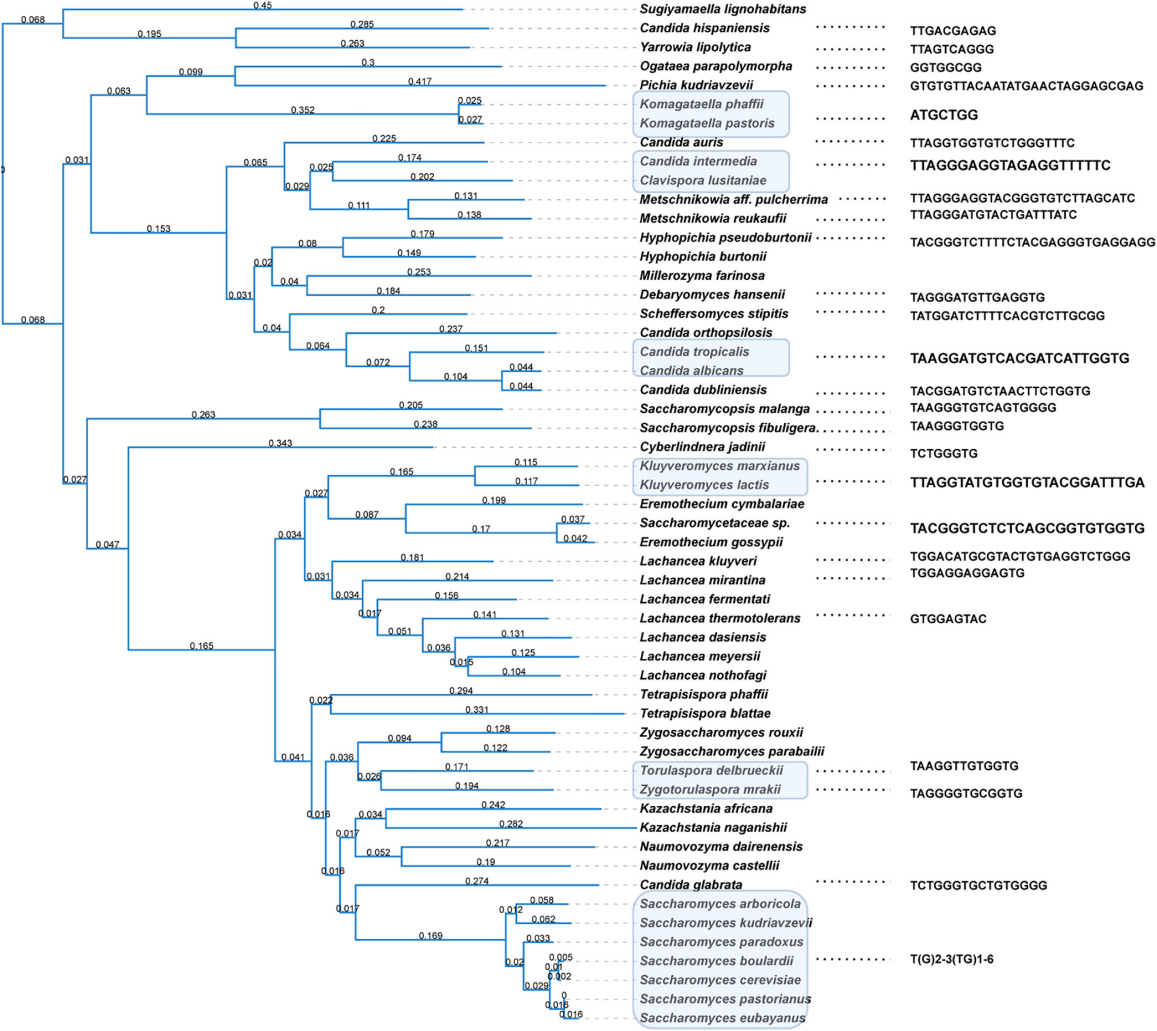
Overview of the telomeric motif sequences of fungi in Saccharomycotina. Left: maximum likelihood phylogenetic tree of 54 Saccharomycotina fungi constructed using single-copy genes. Right: telomeric motif sequences of those fungi. The numbers on the clade indicate the evolutionary distance. The species shaded in blue share the same telomeric motif sequence.

Some species have telomeric motif sequences distinct from the consensus sequence of their genus. Candida dubliniensis lacks the *Candida*-type motif GGATGTCACGATCATTGGTGTAA and instead carries GGATGTCTAACTTCTGGTGTAC (Fig. 2). The pairwise sequence alignment of these two sequences indicated that the two motifs are very similar. Similar observations were also made in the genera *Clavispora*, *Metschnikowia*, and *Saccharomycopsis*. TTAGGGAGGTACTGATGTTCT was found in Candida intermedia and Clavispora lusitaniae, while Candida auris, a yeast in the same genus, *Clavispora*, has the motif TTAGGTGGTGTCTGGGTTTC. The detection of two yeasts in the genus *Metschnikowia* suggested a considerable divergence in telomeric motif sequences. TTAGGGAGGTACGGGTGTCTTAGCATC was identified in *Metschnikowia* aff. *pulcherrima*. TTAGGGATGTACTGATTTATC was discovered in *Metschnikowia reukaufii*. In the genus *Saccharomycopsis*, TAAGGGTGGTG and TAAGGGTGTCAGTGGGG were found in Saccharomycopsis fibuligera and Saccharomycopsis malanga, respectively.

**FIG 3:**
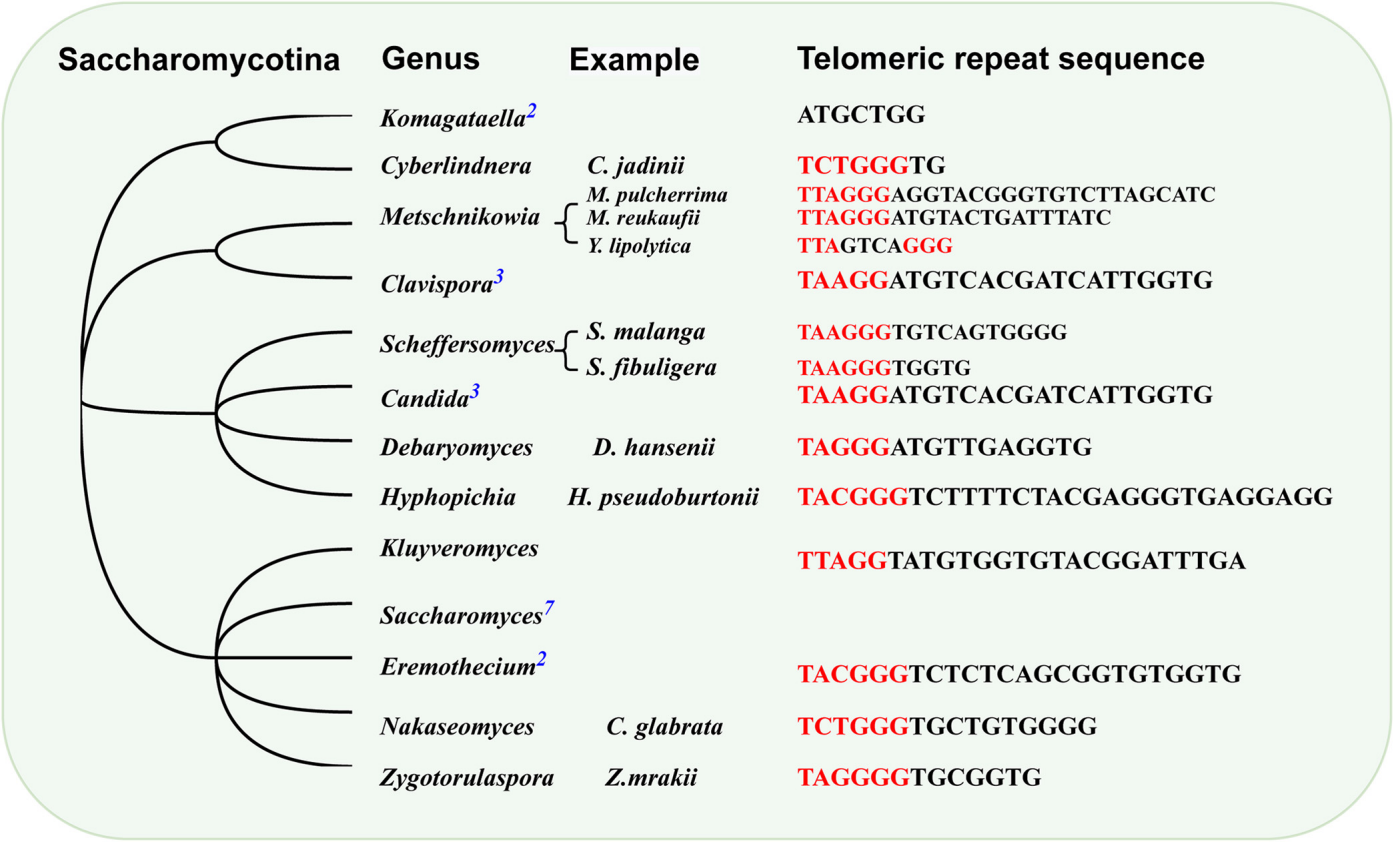
The telomeric repeat sequences of fungi in Saccharomycotina. The number on the genus name indicates the number of species in the genus that contain the corresponding telomeric repeat sequence. The red part of the telomeric repeat sequence is the variant of TTAGGG, indicating that the ancestor of Saccharomycotina may carry TTAGGG.

Notably, although these telomeric motif sequences in the subphylum Saccharomycotina are divergent and irregular, most seem to be variants of the vertebrate-type TTAGGG (Fig. 3).

### Variation analysis in telomere sequence.

In addition to identifying telomeric motif sequences, TelFinder can be used to analyze variations in telomere sequences within a species, including base changes, insertions, and deletions. Since it is difficult to distinguish whether a telomere-like sequence was derived from multiple base-change events and many insertions accompanied by deletion events, only single-base variation (SV) in one telomeric motif sequence is considered. Here, variations in the telomere sequences of Homo sapiens (GCF_009914755.1), Arabidopsis thaliana (GCA_020911765.1), and Yarrowia lipolytica (GCA_014490615.1) are shown as examples of animals, plants, and fungi. The beginning and end of one chromosome are defined as the “left end” and the “right end,” respectively. Both ends can provide information about telomeric motifs and variations. The telomeric motif sequences at both ends are reverse complementary sequences, suggesting that telomeres at each end of the chromosome are in opposite directions. In addition, the telomeric sequence at the left end is always C-rich (such as CCCTAA) and that at the right end is G-rich (such as TTAGGG). This finding held true across all vertebrates and fungi in Ascomycota that we examined.

**FIG 4:**
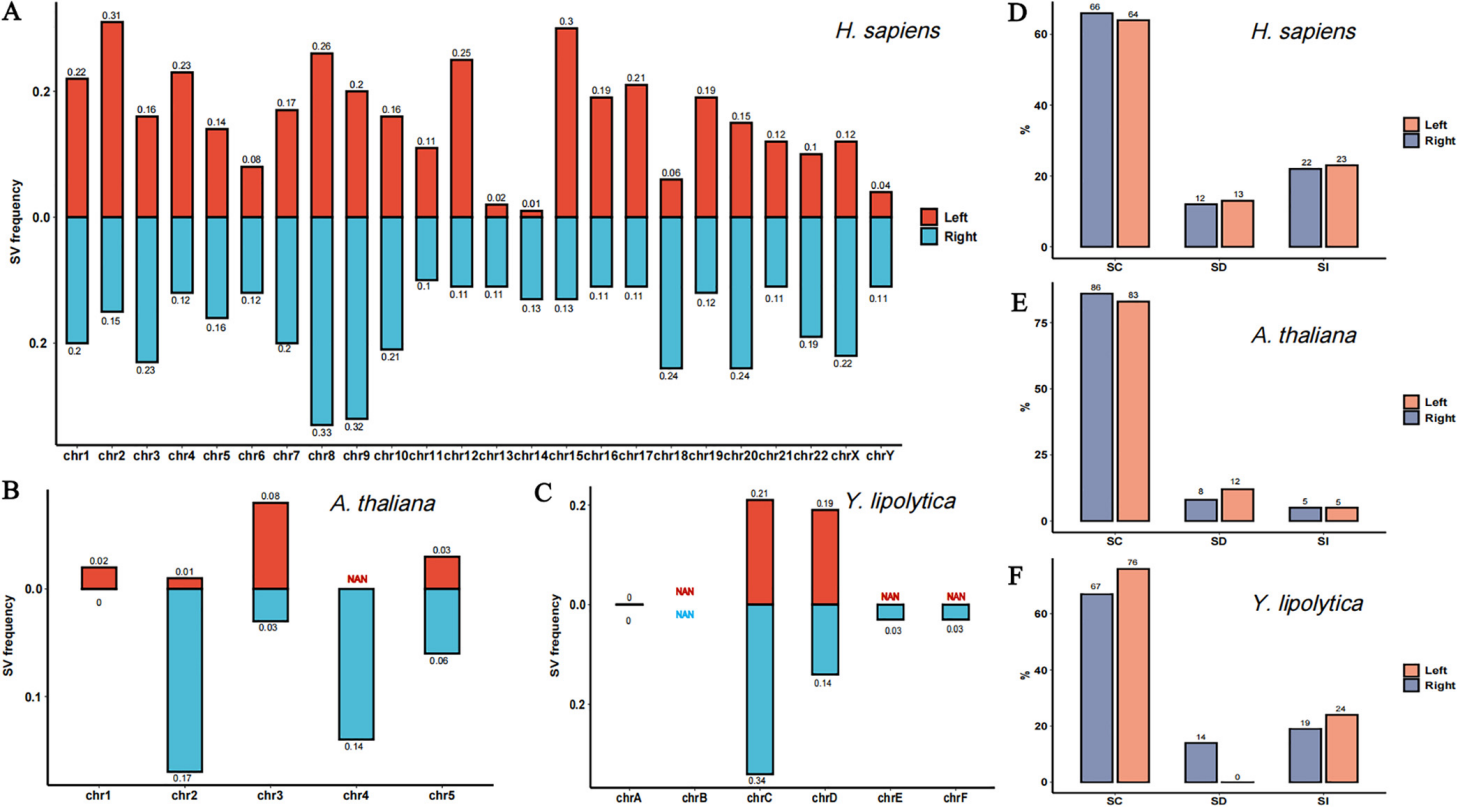
The SV frequency of each chromosome with single-base variations in H. sapiens (A), *A. thaliana* (B), and Y. lipolytica (C). The *x* axis denotes the chromosome, and the *y* axis represents the SV frequency. NAN, the telomere is too short to detect at the corresponding chromosomal end. The proportions of three types of SV (SD, SI, and SC) in the telomeres of H. sapiens (D), *A. thaliana* (E), and Y. lipolytica (F). The *y* axis shows the percentage of each type of SV.

The maximum assembled lengths of H. sapiens, *A. thaliana*, and Y. lipolytica telomeres are approximately 6,389 nt, 4,480 nt, and 663 nt, respectively (see Table S3 in the supplemental material), including the variations. The SV frequency of H. sapiens ranges from 0.01 to 0.31 at the left end and from 0.1 to 0.33 at the right end (Fig. 4A). Chromosomes 2, 8, 9, and 15 have a higher SV frequency (>0.3). SV frequency in the telomeres of *A. thaliana* ranges from 0.01 to 0.08 at the left end and 0 to 0.17 at the right end (Fig. 4B), which is lower than that in H. sapiens. The telomere sequences assembled at the left end of chromosome 4 are too short to detect; thus, the SV frequency cannot be calculated. The telomeres assembled in Y. lipolytica were very short, and no telomeres were assembled at either end of chromosome B. The SV frequency ranged from 0 to 0.21 at the left end and from 0 to 0.34 at the right end (Fig. 4C). In all sample species, the proportions of each SV to the total SV count between the left and right ends are very close. In addition, SC is the most frequent SV in H. sapiens, *A. thaliana*, and Y. lipolytica (Fig. 4D, E, and F).

**FIG 5:**
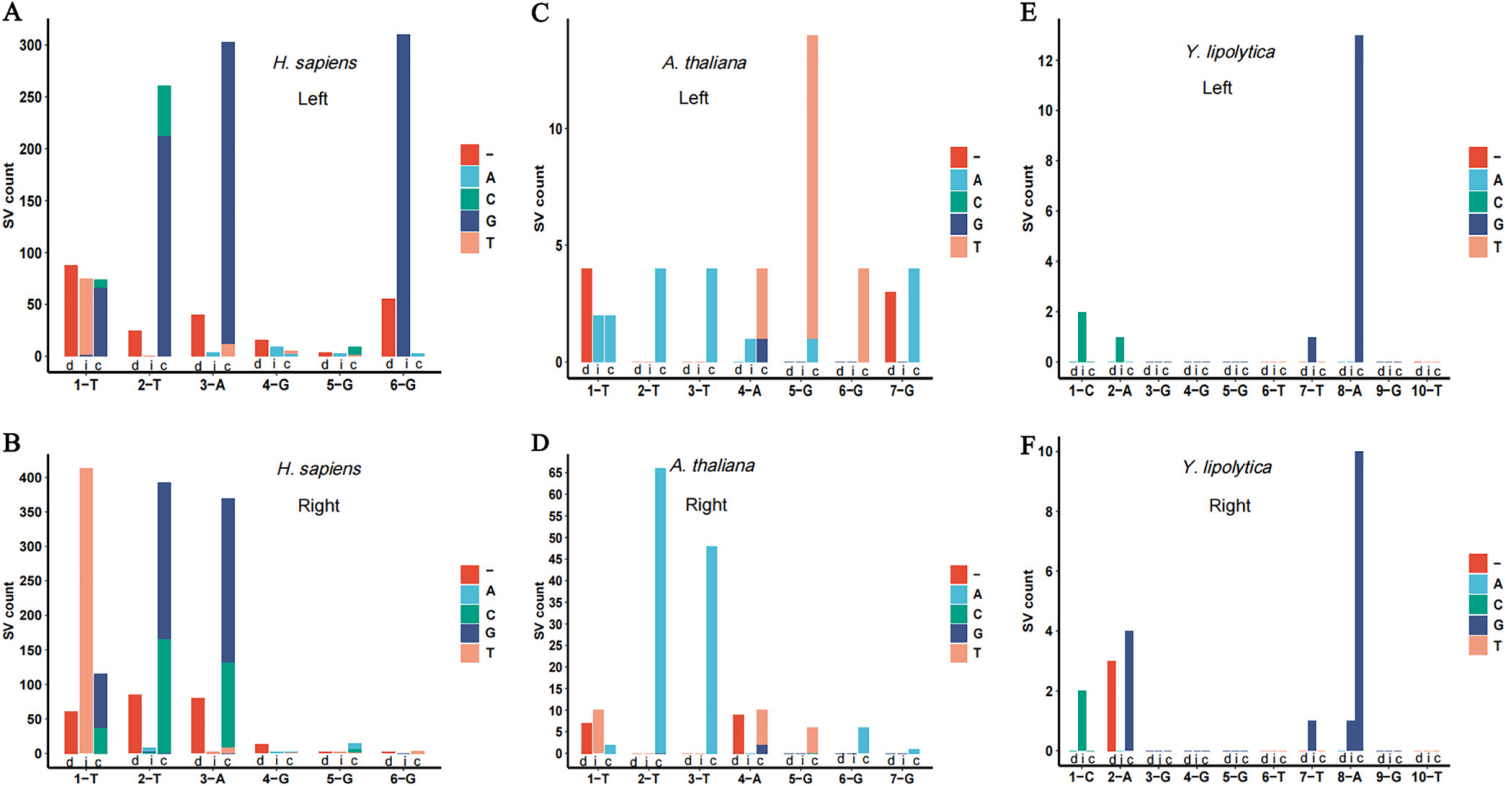
Statistics of the single-base variation (SV) pattern of both ends of the telomere motif in three species (from left, H. sapiens, *A. thaliana*, and Y. lipolytica). The *x* axis represents the site and the corresponding base of the reference telomeric motif sequence. Each site has three bars, as follows: “d,” “i,” and “c,” indicating “deletion,” “insertion,” and “base change,” respectively.

To determine which position is prone to variation, we also compared the SV count at each position of the telomeric motif sequence. The comparison was made at both ends, and the motifs at both ends are reciprocal complementary sequences. For clarity and simplification, the motifs at the two ends are written in the same direction (right end) in Fig. 5. At the left end of the H. sapiens telomere, G insertion and mutation to G are very common, leading to more consecutive Gs, resulting in TTAGGGG or TTGGGG arising from TTAGGG (Fig. 5B). Correspondingly, the insertion of T is most frequent at the right end (Fig. 5A). The telomeric motif sequence of *A. thaliana* is similar to that of H. sapiens, with a T inserted. However, at the right end, the mutation from T to A is prevalent, leading to the change from TTTAGGG to TTAAGGG or TATAGGG (Fig. 5D). A mutation from G to T at site 5 occurred frequently at the left end, yielding TTTATGG from TTTAGGG (Fig. 5C). The telomere assembly of Y. lipolytica is relatively short; thus, few variations were observed. A mutation from A to G at site 8 was frequent at both ends, leading to CAGGGTTGGT arising from CAGGGTTAGT (Fig. 5E and F). It was found that this site is prone to A-to-G mutations.

As seen from the above example, determining which sites have a mutation is challenging. For example, if TTTTAGGG was observed in *A. thaliana*, it would have been difficult to determine at which position the T was inserted. The same situation also occurs in TTAGGG (T deleted), TTTAGGGG (G inserted), and TTTAGG (G deleted). We can only use hints provided by sequence alignment to address this problem at present. To be clear, this is a compromise, as sequence alignment cannot determine the actual position of variation in this case and can only provide the possible sites with variations.

## DISCUSSION

Telomeres have attracted increasing attention due to their prominent role in the cell cycle and senescence (23). However, a practical computational tool for detecting telomeric motif sequences was lacking. This issue prompted us to develop TelFinder for the *de novo* identification of telomeric motif sequences from available genomic data.

By scoring kmers for length and repeat times, the kmer with the highest score on each chromosome can be selected as the potential telomeric motif sequence by TelFinder. Regarding the scoring principle, it is considered that simply multiplying the kmer length and its repeat number means that they are equally important, which may not reflect the underlying biological mechanism. Therefore, gradient coefficients (ranging from 0.1 to 1) were added to the two factors in the scoring formula within the left end of *A. thaliana* and Y. lipolytica. However, the detection results show that the accuracy was not sensitive to the coefficient (see Table S4 in the supplemental material). Thus, we scored a given kmer by directly multiplying the kmer length and repeat times.

We evaluated the telomere content information available in the Telomerase Database to validate the power of TelFinder. Among the species in the database, 43 had genomic data with an assembly level higher than the chromosome. The telomeric repeats were detected successfully in 40 species, and 39 of these detections were correct. These results show that the performance of TelFinder is excellent.

Rather than providing a kmer list based on the repeat times, candidate sequences are evaluated by TelFinder based on reliability by considering both Value and suppChr. The calculation of these two parameters is described in detail in the Materials and Methods section. The motif length, number of motif repeats, and chromosome support were comprehensively considered in the scoring and ranking. In addition, if one of the candidates was a substring of another candidate, we also provided the corresponding penalty points. Therefore, the ranking is more reliable than only considering repeat times, as is the case with SERF. However, the recommendations are somewhat subjective when the candidates have similar Value and suppChr. It is suggested that combining techniques, such as cytological validation with fluorescence *in situ* hybridization (FISH) (14), should be used when the detection result is ambiguous. However, the telomeric repeat sequences of 35 out of 134 fungi were not determined, although they have sufficiently assembled genomic data. To explore the reason for the failure, we rechecked the sequences at the chromosomal termini of these species by eye, and the reasons for the failure can be roughly divided into 2 types (see Table S5 in the supplemental material). One is the “variable telomeric repeat sequence,” accounting for 8 fungi. In these cases, the variations between the kmer candidate and the telomeric repeats are identified as gap sequences, causing a considerable penalty. This process leads to lower scores for the short kmers, while the “winning” kmer represents the combination of at least two repeats. Despite this limitation, TelFinder works for the vast majority of species.

One of two critical parameters is the sequence length to be searched. The default length is 1,000 nt (1,000 nt at the end of a chromosome), and users can adjust the length as needed. A long search range is unnecessary when determining telomeric motif sequences. In variation analyses, the search needs to be extended to cover all the telomere sequences that have been assembled. Another critical parameter is the range of kmer lengths. Since all known telomeric motifs fall between 5 and 30 nt (9, 10, 24), we set this range the default. As this range increases, the computation time required also increases. Therefore, users can choose an appropriate range according to their needs.

In contrast to those in other eukaryotes, the telomeric motif sequences of some fungi are diverse in base composition and length (10), making the evolution of fungal telomeres more intriguing. Previous studies have shown that the telomeric motif sequences of yeast in Saccharomycotina are dramatically divergent (10, 25). However, as many telomeric repeat sequences of this branch were newly identified, these seemingly irregular and divergent motif sequences could be variants of TTAGGG or could contain variants of TTAGGG (Fig. 3). From an evolutionary perspective, telomeric motif sequences in the subphylum Saccharomycotina seem to share an ancestor with vertebrates (TTAGGG). The high diversity of telomeric repeat sequences in Saccharomycotina could arise from the rapid evolution of telomerase RNA in this clade, which is faster than in others. This hypothesis requires further exploration for validation.

In addition to identifying the telomeric repeat sequence from genomic data, TelFinder can be used to conduct variation analyses in telomere sequences. The SV frequencies of distinct chromosome ends of the same species are quite different. Determining whether the SV frequency is a characteristic specific to chromosomes, cell state, or species is worth considering. The SV frequency and length of assembled telomeres on each chromosome of H. sapiens, *A. thaliana*, and Y. lipolytica were compared, and the SV frequency did not correlate with the length. In addition, the length of the assembled telomeres of H. sapiens and *A. thaliana* is similar, but the SV frequency of H. sapiens is generally higher than that of *A. thaliana*. This observation suggests that the SV frequency is species specific to some extent. Moreover, a single-base change (SC) is the most common SV type in H. sapiens, *A. thaliana*, and Y. lipolytica. H. sapiens tends to have insertions that form TTAGGGG. The T-to-A mutation occurs at a high frequency in *A. thaliana*. Given that telomere assembly lengths and compositions are similar between H. sapiens and *A. thaliana*, it is assumed that the preference for variation type is also species specific. Y. lipolytica mostly has A-to-G mutations at site 8. Thus, the locations prone to variation vary across species. Notably, “variation” here is a definition provided for convenience in the statistics and description. It does not indicate that the specific variations are genuinely due to deletions and insertions. Variations in telomere sequences may result from errors in telomere synthesis or from recombination and mutation after synthesis. The simplest explanation could be a mutation in the telomerase RNA’s template region and a template usage change (26). The deletion of T or G in the telomeres of green algae can result from this occurrence. In addition, an inaccurate anchor site of the template in telomerase RNA when annealing can also cause variations in telomeres. It has been proposed that after the +1 elongation in Schizosaccharomyces pombe, telomerase can copy the rest of the template or translocate the template along the telomere (27, 28). Successive translocation and elongation can explain the introduction of multiple guanosines before the consensus sequence. These abnormal variations in telomeres can affect their structure and stability. Detecting the variations using TelFinder can undoubtedly be of substantial importance for quickly exploring the mechanisms and implications of telomere variation.

Although the telomere composition can be determined quickly by TelFinder and only genomic data with telomere information included are needed, our analysis demonstrates that TelFinder has limitations. For example, TelFinder cannot be applied for genomes without assembled telomeres. However, TelFinder is much quicker and more convenient than experimental methods and improves the utilization of genomic data. Although complete genomes are optimal for TelFinder, it is also possible to find potential telomeric repeat sequences in incomplete genomes if the telomere is included in the assembly. In addition, variable telomeric repeat sequences cannot be identified because the variable base would be treated as a gap, leading to an unexpected penalty. Moreover, as the boundary of the telomere is challenging to determine and the assembled telomere is only a part of the complete telomere, an analysis of the variation in the telomere sequence inevitably has bias. It is not yet known the extent to which this bias affects the variation type preferences in telomeres, as related information is limited. If later technologies provide longer telomeres, more accurate information about variations in the telomeres will be obtained. This improvement will have considerable implications for the study of telomeres. Another factor that may affect the SV frequency is base quality in the telomere region. Since read quality is provided in rarely assembled genomes, it is recommended that users obtain variation information from high-quality raw reads. However, with this option, the SV frequency represents the average result across the genome. In contrast, the SV frequencies among the chromosomes can be compared with the assembled genome. Therefore, users can make decisions according to the requirements and available data. In summary, we believe our work will stimulate further studies aimed toward obtaining a deeper understanding of telomere variation and its implications.

## MATERIALS AND METHODS

### Principle of the algorithm.

Telomeres are characterized by repeated sequences at the ends of chromosomes. Therefore, the most likely telomeric repeat sequence can be detected by identifying the sequence most frequently repeated at the chromosomal terminal. The pipeline of TelFinder is shown in Fig. 1A.

TelFinder is implemented in the Python language and takes assembled genomes in Fasta format or raw read data in Fastq format as the input. The workflow is described as follows (Fig. 1A). Whether the users input Fasta or Fastq files, the first step of TelFinder is to extract the sequences where the telomeres are present. If the assembled genome is input, the sequences that need to be extracted are the *n* nt in the chromosomal terminal. We set *n* as 1,000 by default. The linear chromosome has two ends, and we defined the beginning of the genome as the left end and the other end as the right end. Therefore, each round detects only a specified end. The continuous “N” at the end of the chromosome is not included in this length. This parameter can be adjusted on demand. Users can also enter raw read data if telomeres are absent from the assembled genome. The input read data will be mapped to the reference genome in the preprocessing step. As the telomeres are not assembled in the reference genome, telomeric reads can be among the unmapped reads. The next steps are the same in both modes. In the second step, a sliding window divides the extracted sequences into equal-length segments (kmers). The window size represents the length of the expected telomeric motif sequence. Since the known telomeric motif sequence length varies from 5 to 30 nt, the default window is set to this range. The length of the query telomeric motif sequence is usually unclear; thus, the length of the assigned range is tested individually. Each kmer is scored by considering the kmer length and repeat times, as follows: S_kmer_ = kmer length × repeat times. When two kmers at one chromosomal terminal are compared, the difference in score S_kmer_ between two kmers needs to be calculated, namely, Value_kmer1-kmer2_ = S_kmer1_ − S_kmer2_. If Value_kmer1-kmer2_ if >0, then kmer1 is the winner. All types of kmers on one chromosome end are compared in pairs, and the kmer with the most win times is the most recommended candidate on that chromosomal end. When kmer1 is a substring of kmer2 (for example, TTAGG is a substring of TTAGGG), an extra penalty needs to be assigned to kmer1. In this case, Value_kmer1-kmer2_ = (S_kmer1_ − S_kmer2_) − repeat times (kmer1 as substring) × kmer1 length. Finally, the number of chromosomes that most recommend the kmer, namely, suppChr, are counted. The candidate kmer with the highest suppChr value will be considered the most likely telomeric repeat sequence.

### Genome analyses.

Telomeric repeat sequence detection was performed on all species whose telomere compositions are known in the Telomerase Database, including vertebrates, invertebrates, fungi, plants, and ciliates, as listed in Table S1.

Telomeric repeat sequence searching was also performed on fungi (Table S2) with an assembly level higher than the chromosome in the NCBI genome database. The phylogenetic relationship of the fungi in Saccharomycotina was constructed using single-copy core genes by OrthoFinder2 (29).

### Analysis of variations in the telomere sequence.

Variation analysis can be performed using either the assembled genome or raw reads as the input. For both types of input, all gap sequences between two telomeric repeat sequences were extracted. Then, each gap sequence was aligned with the telomeric repeat sequence as a reference by pairwise sequence alignment using MAFFT (30). The parameters for sequence alignment using MAFFT are “clustalout –textmatrix matrix.txt.” Since the default scoring matrix treats a single-base insertion as a substitution and deletion at the end of the sequence, a manually modified scoring matrix is used in the sequence alignment. In detail, we set the mismatch penalty as −2. SVs were counted on both ends of all chromosomes, including single-base changes, deletions, and insertions (SC, SD, and SI, respectively). The SV frequency of each chromosome was calculated using equation 1, where “total motif count” refers to the count of typical motifs and motifs with SV.

(1) $$\text{SV frequency}=\frac{\text{SV count}}{\text{total motif count}}$$

The proportion of each type of SV to the total SV is calculated using equations 2, 3, and 4 as follows:

(2) $$\text{SC\%}=\frac{\text{SC count}}{\text{SV count}}$$

(3) $$\text{SD\%}=\frac{\text{SD count}}{\text{SV count}}$$

(4) $$\text{SI\% }=\frac{\text{SI count}}{\text{SV count}}$$

It is worth noting that only the average SV frequency for the entire genome was estimated when the raw reads were used as the input.

### Data availability.

All data relevant to the study are included in the article or uploaded as supplementary information. The script used is available online at https://github.com/bio-tombow/TelFinder.
