# Correction for Sun et al., “Large-Scale Detection of Telomeric Motif Sequences in Genomic Data Using TelFinder”

**DOI:** 10.1128/spectrum.02777-23

**Published:** 2023-10-13

**Authors:** Qing Sun, Hao Wang, Shiheng Tao, Xuguang Xi

## AUTHOR CORRECTION

Volume 11, no. 2, e03928-22, 2023, https://doi.org/10.1128/spectrum.03928-22.

Page 5, line 23: “TAAGGATGTCACGATCATTGGTG was detected in *Candida tropicalis* and *Candida albicans*,” should be “TACGGATGTCTAACTTCTTGGTG was detected in *Candida albicans*.”

Page 5, line 39: “*Eremothecium gossypii*” should be “*Eremothecium cymbalariae*.”

Page 5, line 40: “This motif was also identified in another species in the genus *Eremothecium*, namely, *Eremothecium cymbalariae*.” should read "A similar motif, TCTCTCAGCGGTGTGGTGTATGGG, was identified in *E. gossypii*, another species in the genus *Eremothecium*.”

Page 5, line 46: “*E. cymbalariae*” should be “*E. gossypii*.”

Page 6, Fig. 2: The telomeric motif sequences of *Candida albicans* and *Kluyveromyces lactis* were mislabeled. The correct Fig. 2 is shown in this author correction. The corresponding motif is also corrected in the revised Table S1 in this author correction.

**Fig 2:**
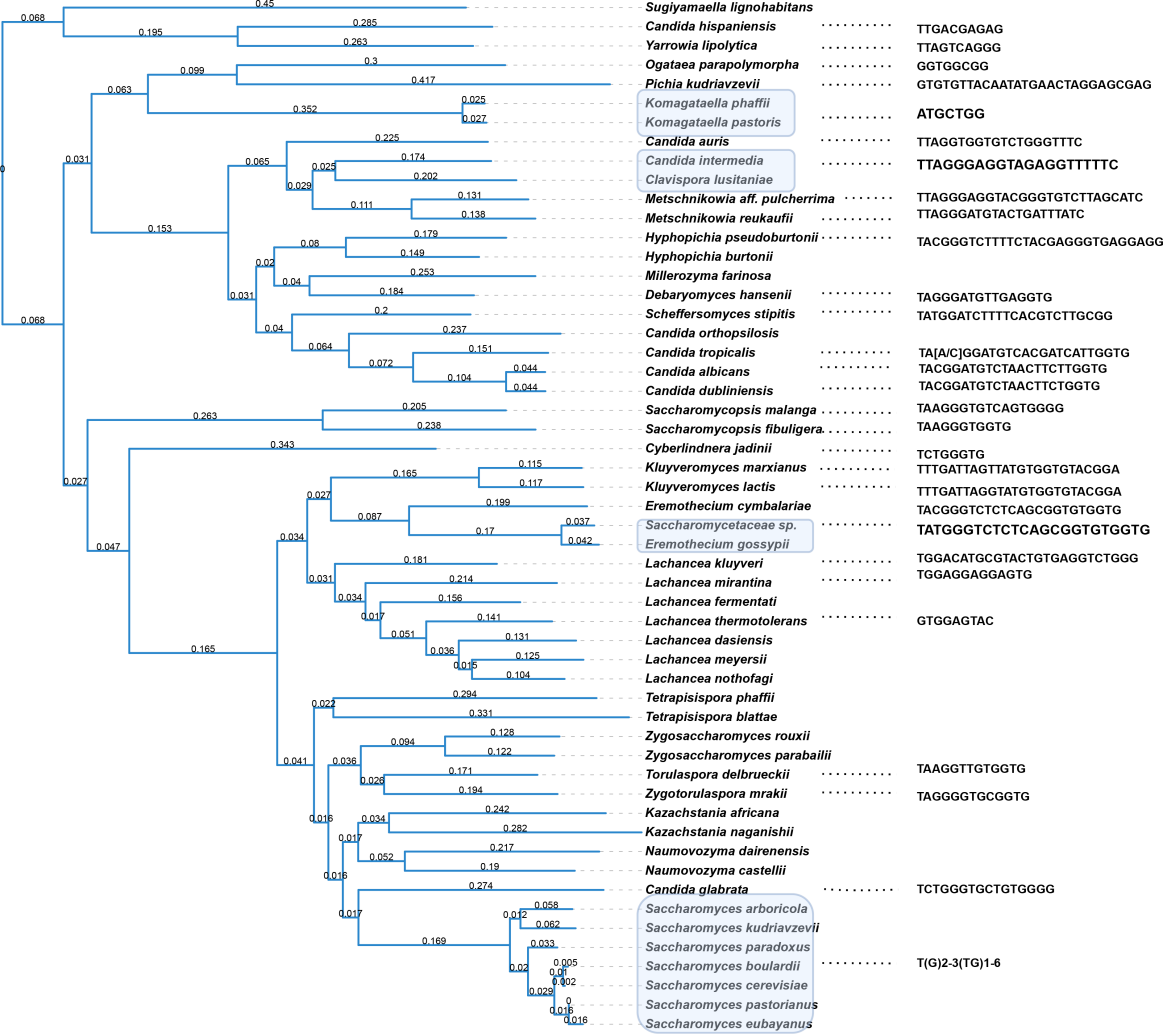


Page 7, Fig. 3: The genus names of *Saccharomycopsis malanga* and *Saccharomycopsis fibuligera* were mislabeled. The correct Fig. 3 is shown in this author correction.

**Fig 3:**
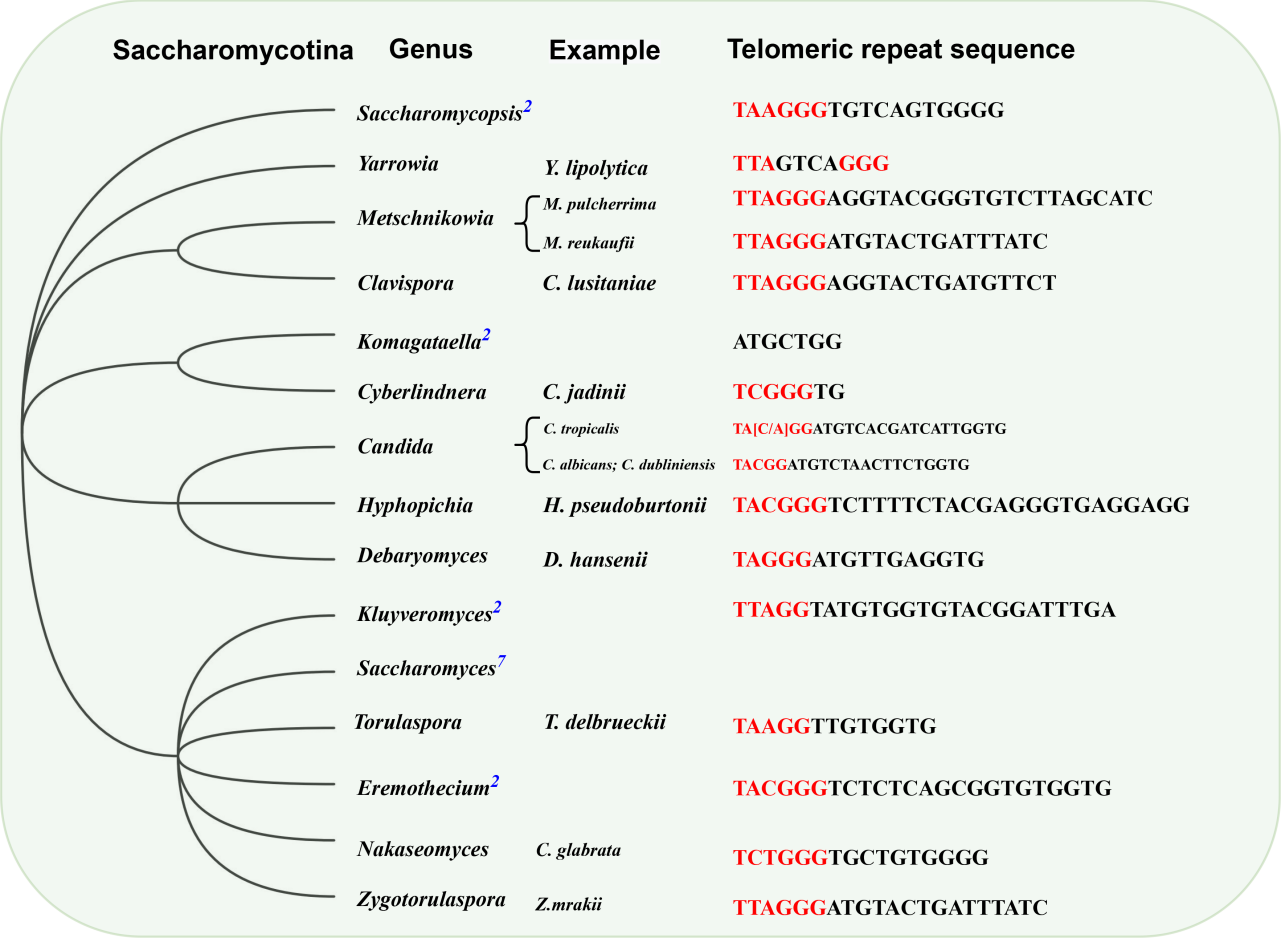


Correction of the information above does not change the conclusions of this paper.

## SUPPLEMENTAL MATERIAL

Supplemental material spectrum.02777-23-s0001.docx Supplemental material for published article; Table S1 is revised

ASM does not own the copyrights to Supplemental Material that may be linked to, or accessed through, an article. The authors have granted ASM a non-exclusive, world-wide license to publish the Supplemental Material files. Please contact the corresponding author directly for reuse.

