# Supplementary material for: Large-Scale Detection of Telomeric Motif Sequences in Genomic Data Using TelFinder
Source: Microbiol Spectr. 2023 Feb 27;11(2):e03928-22. doi: 10.1128/spectrum.03928-22 (PMC10100673; doi:10.1128/spectrum.03928-22)
Supplement: Supplemental file 1 — Supplemental material. Download spectrum.03928-22-s0001.pdf, PDF file, 0.2 MB [file spectrum.03928-22-s0001.pdf]

Table S1: Application of TelFinder to a reference dataset in Telomerase Database

	species	Sequences	References	genomic	TelFinder	Accordance	chr with telomere	telchrN/allchrN	max Tellen
**Invertebrates**	Tribolium castaneum (red flour beetle)	TCAGG	Osanai, M., Kojima, K. K., Futahashi, R., Yaguchi, S., & Fujiwara, H. (2006). Identification and characterization of the telomerase reverse transcriptase of Bombyx mori (silkworm) and Tribolium castaneum (flour beetle). Gene, 376(2), 281–289.	yes	TCAGG	yes	NC_007417.3; NC_007424.3; NC_007425.3	3/11	199
	Tribolium confusum	TCAGG	Mravinac, B., Meštrović, N., Cavrak, V. V., & Plohl, M. (2011). TCAGG, an alternative telomeric sequence in insects. Chromosoma, 120(4), 367–376.	yes	TCAGG	yes	CM032790.1	1/8	373
	Apis mellifera (honey bee)	TTAGG	Sahara, K., Marec, F., & Traut, W. (1999). TTAGG telomeric repeats in chromosomes of some insects and other arthropods. Chromosome research : an international journal on the molecular, supramolecular and evolutionary aspects of chromosome biology, 7(6), 449–460.	yes	TTAGG	yes	NC_037644.1; NC_037640.1; NC_037645.1; NC_037638.1; NC_037639.1; NC_037651.1; NC_037647.1; NC_037653.1; NC_037641.1; NC_037650.1; NC_037643.1; NC_037652.1; NC_037649.1	13/17	11785

	Mamestra brassicae (cabbage moth)	TTAGG	Frydrychová, R., Grossmann, P., Trubac, P., Vítková, M., & Marec, F. (2004). Phylogenetic distribution of TTAGG telomeric repeats in insects. Genome, 47(1), 163–178.	yes	TTAGG	yes	LR990988.1; LR990991.1; LR990992.1; LR990993.1; LR990995.1; LR990996.1; LR990997.1; LR990998.1; LR990999.1; LR991000.1; LR991001.1; LR991003.1; LR991004.1; LR991005.1; LR991006.1; LR991007.1; LR991008.1; LR991009.1; LR991010.1; LR991011.1; LR991012.1; LR991013.1; LR991014.1; LR991015.1; LR991016.1; LR991017.1; LR990987.1	23/32	15850
	Ascaris suum	TTAGGC	Teixeira, M. T., & Gilson, E. (2005). Telomere maintenance, function and evolution: the yeast paradigm. Chromosome research : an international journal on the molecular, supramolecular and evolutionary aspects of chromosome biology, 13(5), 535–548.	yes	TTAGGC	yes	CM024168.1; CM024170.1; CM024188.1; CM024172.1; CM024174.1; CM024190.1	6/25	49704
	Caenorhabditis elegans	TTAGGC	Cangiano, G., & La Volpe, A. (1993). Repetitive DNA sequences located in the terminal portion of the Caenorhabditis elegans chromosomes. Nucleic acids research, 21(5), 1133–1139.	yes	TTAGGC	yes	NC_003279.8; NC_003280.10; NC_003281.10; NC_003282.8; NC_003283.11; NC_003284.9	6/7	845
**Fungi**	Schizosaccharomyces pombe (fission yeast)	G2–8TTAC(A)	Joseph, I., & Lustig, A. J. (2007). Telomeres in meiotic recombination: the yeast side story. Cellular and molecular life sciences : CMLS, 64(2), 125–130.	yes	G2–8TTAC(A)	/	/	/	/

	Saccharomyces cerevisiae (baker's yeast)	T(G)2-3(TG)1-6	McEachern, M. J., & Blackburn, E. H. (1994). A conserved sequence motif within the exceptionally diverse telomeric sequences of budding yeasts. Proceedings of the National Academy of Sciences of the United States of America, 91(8), 3453–3457.	yes	T(G)2-3(TG)1-6	/	/	/	/
	Saccharomyces bayanus	T(G)2-3(TG)1-6	Teixeira, M. T., & Gilson, E. (2005). Telomere maintenance, function and evolution: the yeast paradigm. Chromosome research : an international journal on the molecular, supramolecular and evolutionary aspects of chromosome biology, 13(5), 535–548.	yes	T(G)2-3(TG)1-6	/	/	/	/
	Saccharomyces paradoxus	T(G)2-3(TG)1-6	Teixeira, M. T., & Gilson, E. (2005). Telomere maintenance, function and evolution: the yeast paradigm. Chromosome research : an international journal on the molecular, supramolecular and evolutionary aspects of chromosome biology, 13(5), 535–548.	yes	T(G)2-3(TG)1-6	/	/	/	/
	Saccharomyces castellii	TCTGGG(TG)1-4	Cohn, M., & Blackburn, E. H. (1995). Telomerase in yeast. Science (New York, N.Y.), 269(5222), 396–400.	yes	T(G)2-3(TG)1-6	/	/	/	/
	Saccharomyces kluyveri	GGGTGGACATG CGTACTGTGAG GTCT	Cohn, M., McEachern, M. J., & Blackburn, E. H. (1998). Telomeric sequence diversity within the genus Saccharomyces. Current genetics, 33(2), 83–91.	yes	TCTGGGTGGAC ATGCGTACTGT GAGG	yes	CM000687.1; CM000688.1; CM000689.1; CM000691.1	4/8	598
	Kluyveromyces lactis	ACGGATTTGATT AGGTATGTGGT GT	McEachern, M. J., & Blackburn, E. H. (1994). A conserved sequence motif within the exceptionally diverse telomeric sequences of budding yeasts. Proceedings of the National Academy of Sciences of the United States of America, 91(8), 3453–3457.	yes	TTAGGTATGTG GTGTACGGATT TGA	yes	NC_006037.1; NC_006038.1; NC_006039.1; NC_006040.1; NC_006041.1; NC_006042.1	6/7	708

	Candida albicans	ACGGATGTCTAACTTCTTGGTGT	McEachern, M. J., & Blackburn, E. H. (1994). A conserved sequence motif within the exceptionally diverse telomeric sequences of budding yeasts. Proceedings of the National Academy of Sciences of the United States of America, 91(8), 3453–3457.	yes	TAAGGATGTCACGATCATTGGTG	yes	CP032017.1	1/8	931
	Candida glabrata	CTGGGTGCTGTGGGGT	McEachern, M. J., & Blackburn, E. H. (1994). A conserved sequence motif within the exceptionally diverse telomeric sequences of budding yeasts. Proceedings of the National Academy of Sciences of the United States of America, 91(8), 3453–3457.	yes	TCTGGGTGCTGTGGGG	yes	CP048234.1; CP048230.1; CP048240.1; CP048241.1; CP048236.1; CP048235.1; CP048232.1; CP048238.1; CP048233.1; CP048237.1; CP048231.1; CP048239.1; CP048242.1	13/13	817
	Candida orthopsilosis	GGTTAGGATGTAGACAATACTGC	Gunisova, S. (2009). Identification and comparative analysis of telomerase RNAs from Candida species reveal conservation of functional elements. RNA (New York, N.Y.), 15(4), 546–559.	yes	/	/	/	/	/
	Candida pseudotropicalis	ACGGATTTGATTAGTTATGTGGTGT	McEachern, M. J., & Blackburn, E. H. (1994). A conserved sequence motif within the exceptionally diverse telomeric sequences of budding yeasts. Proceedings of the National Academy of Sciences of the United States of America, 91(8), 3453–3457.	yes	TACGGATTTGATTAGTTATGTGGTG	yes	NC_036025.1; NC_036026.1; NC_036027.1; NC_036028.1; NC_036029.1; NC_036030.1; NC_036031.1; NC_036032.1	8/9	750

	Candida tropicalis	A[C/A]GGATGTC ACGATCATTGG TGT; AAGGATGTCAC GATCATTGGTGT	Gunisova, S. (2009). Identification and comparative analysis of telomerase RNAs from Candida species reveal conservation of functional elements. RNA (New York, N.Y.), 15(4), 546–559. McEachern, M. J., & Blackburn, E. H. (1994). A conserved sequence motif within the exceptionally diverse telomeric sequences of budding yeasts. Proceedings of the National Academy of Sciences of the United States of America, 91(8), 3453–3457.	yes	TAAGGATGTCA CGATCATTGGT G	yes	CP047875.1; CP047869.1; CP047870.1; CP047871.1; CP047872.1; CP047873.1; CP047874.1	7/7	199
	Debaryomyces hansenii	ATGTTGAGGTG TAGGG	Lépingle, A., Casaregola, S., Neuvéglise, C., Bon, E., Nguyen, H., Artiguenave, F., Wincker, P., & Gaillardin, C. (2000). Genomic exploration of the hemiascomycetous yeasts: 14. Debaryomyces hansenii var. hansenii. FEBS letters, 487(1), 82–86.	yes	TAGGGATGTTG AGGTG	yes	NC_006044.2; NC_006048.2	2/8	315
	Ashbya gossypii (Eremothecium gossypii)	GTGTGGTGTAT GGGTCTCTCAG CG	Dietrich, F. S., Voegeli, S. (2004). The Ashbya gossypii genome as a tool for mapping the ancient Saccharomyces cerevisiae genome. Science (New York, N.Y.), 304(5668), 304–307.	yes	TATGGGTCTCT CAGCGGTGTG GTG	yes	NC_005782.2; NC_005783.5; NC_005784.3; NC_005785.6; NC_005786.2; NC_005787.5; NC_005788.4	7/8	672
	Pichia stipitis	GGATCTTTTCAC GTCTTGCGGTA	Jeffries, T. W., Grigoriev, I. V. (2007). Genome sequence of the lignocellulose-bioconverting and xylose-fermenting yeast Pichia stipitis. Nature biotechnology, 25(3), 319–326.	yes	GGATCTTTTCA CGTCTTGCGGT AT	yes	NC_009068.1; NC_009042.1; NC_009043.1; NC_009044.1; NC_009045.1; NC_009046.1; NC_009047.1; NC_009048.1	8/8	558
	Yarrowia lipolytica	GGACGATTG	Teixeira, M. T., & Gilson, E. (2005). Telomere maintenance, function and evolution: the yeast paradigm. Chromosome research : an international journal on the molecular, supramolecular and evolutionary aspects of chromosome biology, 13(5), 535–548.	yes	TCAGGGTTAG	no	CP061012.1; CP061014.1; CP061015.1; CP061016.1; CP061017.1	5/7	663

	Clavispora lusitaniae	TCTTTAGGGAG GTACTGATGT	Gunisova, S. (2009). Identification and comparative analysis of telomerase RNAs from Candida species reveal conservation of functional elements. RNA (New York, N.Y.), 15(4), 546–559.	yes	TTTAGGGAGGT ACTGATGTTC	yes	CP038484.1; CP038485.1; CP038486.1; CP038487.1; CP038490.1	5/8	396
	Aspergillus fumigatus	TTAGGG	Nierman, W. C. (2005). Genomic sequence of the pathogenic and allergenic filamentous fungus Aspergillus fumigatus. Nature, 438(7071), 1151–1156.	yes	TTAGGG	yes	NC_007194.1; NC_007195.1; NC_007196.1; NC_007197.1; NC_007198.1; NC_007199.1; NC_007200.1; NC_007201.1	8/8	112
	Aspergillus oryzae	TTAGGGTCAAC A	Kusumoto, K. I., Suzuki, S., & Kashiwagi, Y. (2003). Telomeric repeat sequence of Aspergillus oryzae consists of dodeca-nucleotides. Applied microbiology and biotechnology, 61(3), 247–251.	yes	TTAGGGTCAAC A	yes	NC_036435.1; NC_036439.1; NC_036441.1; NC_036438.1; NC_036442.1	5/9	98
	Aspergillus nidulans (Emericella nidulans)	TTAGGG	Bhattacharyya, A., & Blackburn, E. H. (1997). Aspergillus nidulans maintains short telomeres throughout development. Nucleic acids research, 25(7), 1426–1431.	yes	TTAGGG	yes	BN001302.1; BN001306.1; BN001307.1; BN001305.1; BN001308.1	5/8	157
	Histoplasma capsulatum	TTAGGG	Woods, J. P., & Goldman, W. E. (1992). In vivo generation of linear plasmids with addition of telomeric sequences by Histoplasma capsulatum. Molecular microbiology, 6(23), 3603–3610.	yes	TTAGGG	yes	CP069114.1; CP069109.1; CP069110.1; CP069111.1; CP069116.1; CP069112.1	6/8	198
	Magnaporthe grisea (rice blast fungus)	TTAGGG	Teixeira, M. T., & Gilson, E. (2005). Telomere maintenance, function and evolution: the yeast paradigm. Chromosome research : an international journal on the molecular, supramolecular and evolutionary aspects of chromosome biology, 13(5), 535–548.	yes	TTAGGG	yes	NC_044975.1	1/3	105

	Podospora anserina	TTAGGG	Javerzat, J. P., Bhattacherjee, V., & Barreau, C. (1993). Isolation of telomeric DNA from the filamentous fungus Podospora anserina and construction of a self-replicating linear plasmid showing high transformation frequency. Nucleic acids research, 21(3), 497–504.	yes	TTAGGG	yes	CM030332.1; CM030333.1; CM030335.1; CM030337.1	4/8	120
	Neurospora crassa	TTAGGG	Schechtman M. G. (1990). Characterization of telomere DNA from Neurospora crassa. Gene, 88(2), 159–165.	yes	TTAGGG	yes	NC_026502.1; NC_026503.1; NC_026504.1; NC_026505.1; NC_026506.1; NC_026507.1; NC_026501.1	7/8	309
	Cryptococcus neoformans (Filobasidiella neoformans)	TTA(G)4-6	Edman J. C. (1992). Isolation of telomerelike sequences from Cryptococcus neoformans and their use in high-efficiency transformation. Molecular and cellular biology, 12(6), 2777–2783.	yes	TTAGGGGG	yes	NC_006684.1; NC_006686.1; NC_006691.1; NC_006692.1; NC_006693.1; NC_006694.1; NC_006679.1; NC_006680.1; NC_006681.1; NC_006682.1; NC_006683.1; NC_006670.1; NC_006685.1; NC_006687.1	14/14	93
	Encephalitozoon cuniculi	G[A/G]GCCT[C/T]CT; GAGCCTTGTTT; GAGACGCAGTG TTGCCAGGATG	Peyret, P., Katinka, M. D. (2001). Sequence and analysis of chromosome I of the amitochondriate intracellular parasite Encephalitozoon cuniculi (Microspora). Genome research, 11(2), 198–207.	yes	GGTTATGTGT	no	NC_003242.2	1/11	70
**Amoeba**	Dictyostelium discoideum	A(G)1-8	Emery, H. S., & Weiner, A. M. (1981). An irregular satellite sequence is found at the termini of the linear extrachromosomal rDNA in Dictyostelium discoideum. Cell, 26(3 Pt 1), 411–419.	yes	/	/	/	/	/

**Plants**	Solanum lycopersicum (tomato)	TT[T/A]GGG	Ganal, M. W., Lapitan, N. L., & Tanksley, S. D. (1991). Macrostructure of the tomato telomeres. The Plant cell, 3(1), 87–94.	yes	TTTAGGG	yes	OU640346.1; OU640347.1; OU640349.1; OU640351.1; OU640353.1; OU640354.1; OU640344.1; OU640345.1; OU640352.1	9/12	48474
	Arabidopsis thaliana (thale cress)	TTTAGGG	Richards, E. J., & Ausubel, F. M. (1988). Isolation of a higher eukaryotic telomere from Arabidopsis thaliana. Cell, 53(1), 127–136.	yes	TTTAGGG	yes	CP087126.2; CP087127.2; CP087128.1; CP087129.2; CP087130.2	5/5	4480
**Other Protists**	Plasmodium falciparum (human parasite)	TT[T/C]AGGG	Vernick, K. D., & McCutchan, T. F. (1988). Sequence and structure of a Plasmodium falciparum telomere. Molecular and biochemical parasitology, 28(2), 85–94.	yes	TT[T/C]AGGG	yes	NC_004325.2; NC_037280.1; NC_000521.4; NC_004318.2; NC_004326.2; NC_004327.3; NC_004328.3; NC_004329.3; NC_004330.2; NC_037281.1; NC_037282.1; NC_004331.3; NC_037283.1	13/15	4731
	Plasmodium berghei (rodent parasite)	TT[T/C]AGGG	Ponzi, M., Pace, T., Dore, E., & Frontali, C. (1985). Identification of a telomeric DNA sequence in Plasmodium berghei. The EMBO journal, 4(11), 2991–2995.	yes	TT[T/C]AGGG	yes	NC_036159.2; NC_036160.2; NC_036161.2; NC_036162.2; NC_036163.2; NC_036166.2; NC_036168.2; NC_036169.2	8/16	732
	Theileria annulata	TTTTAGGG	Sohanpal, B. K., Morzaria, S. P., Gobright, E. I., & Bishop, R. P. (1995). Characterisation of the telomeres at opposite ends of a 3 Mb Theileria parva chromosome. Nucleic acids research, 23(11), 1942–1947.	yes	TTTAGGG	no	NC_011100.1; NC_011098.1; NC_011129.2	3/4	1154

	Cryptosporidium parvum	TTTAGG	Liu, C., Schroeder, A. A., Kapur, V., & Abrahamsen, M. S. (1998). Telomeric sequences of Cryptosporidium parvum. Molecular and biochemical parasitology, 94(2), 291–296.	yes	TTTAGG	yes	NC_006981.1; NC_006982.1; NC_006983.1; NC_006984.1; NC_006985.1	5/8	2647
	Giardia lamblia	TTAGG	Morrison, H. G., McArthur, A. G., Gillin, F. D. (2007). Genomic minimalism in the early diverging intestinal parasite Giardia lamblia. Science (New York, N.Y.), 317(5846), 1921–1926.	yes	TAGGG	no	NC_051856.1; NC_051858.1; NC_051859.1	3/5	1772
	Giardia intestinalis	TAGGG	Le Blancq, S. M., Kase, R. S., & Van der Ploeg, L. H. (1991). Analysis of a Giardia lamblia rRNA encoding telomere with [TAGGG]n as the telomere repeat. Nucleic acids research, 19(20), 5790.	yes	TAGGG	yes	NC_051856.1; NC_051858.1; NC_051859.1	3/5	1772
	Leishmania major	TTAGGG	Teixeira, M.T., and Gilson, E. (2005). Telomere maintenance, function and evolution: the yeast paradigm. Chromosome Res 13, 535–548. 10.1007/s10577-005-0999-0.	yes	TTAGGG	yes	NC_004916.2; NC_007245.2; NC_007246.2; NC_007247.2; NC_007248.2; NC_007250.2; NC_007251.2; NC_007252.2; NC_007253.2; NC_007254.2; NC_007255.2; NC_007256.2; NC_007257.2; NC_007258.2; NC_007259.2; NC_007260.2; NC_007261.2; NC_007262.2; NC_007263.2; NC_007264.2; NC_007265.2; NC_007266.2; NC_007267.2; NC_007268.2; NC_007269.2; NC_007270.2; NC_007271.2; NC_007272.2; NC_007273.2; NC_007285.2;	33/36	1321

							NC_007286.2;<br>NC_007284.2;<br>NC_007287.2		
	Trypanosoma brucei	TTAGGG	Blackburn, E. H., & Challoner, P. B. (1984). Identification of a telomeric DNA sequence in Trypanosoma brucei. Cell, 36(2), 447–457.	yes	TTAGGG	yes	NC_008409.1;<br>NC_007283.1	2/10	482

Table S2: Detect telomeric motif sequence of fungi by TelFinder

species	abbr.	motif	len	subphylum	IFdetection	chr with telomere	telchrN/allchrN	max Tellen
Peltaster fructicola	PFA	TAGGG	5	Pezizomycotina	yes	CP051139.1;CP051140.1;CP051141.1;CP051142.1;CP051143.1	5/5	119
Zymoseptoria tritici	ZTR	TTAGGG	6	Pezizomycotina	yes	CM009548.1;CM009544.1;CM009545.1;CM009546.1;CM009541.1;CM009552.1;CM009550.1;CM009539.1;CM009551.1;CM009554.1;CM009547.1;CM009542.1;CM009553.1;CM009556.1;CM009540.1;CM009549.1;CM009543.1;CM009555.1	18/18	270
Cercospora sojina	CSO	/	0	Pezizomycotina	no	/	/	/
Cercospora beticola	CBT	/	0	Pezizomycotina	no	/	/	/
Venturia effusa	VEF	TTAGGG	6	Pezizomycotina	yes	CP042199.1;CP042187.1;CP042204.1;CP042192.1;CP042185.1;CP042188.1;CP042194.1;CP042190.1;CP042195.1;CP042196.1;CP042191.1;CP042189.1;CP042203.1;CP042193.1;CP042198.1;CP042200.1;CP042201.1;CP042202.1;CP042197.1;CP042186.1	20/21	168
Pyrenophora teres	PTE	TTAGGG	6	Pezizomycotina	yes	CM017824.1;CM017827.1;CM017826.1;CM017821.1;CM017831.1;CM017825.1;CM017829.1;CM017822.1;CM017830.1;CM017820.1;CM017828.1;CM017823.1	12/12	144
Bipolaris sorokiniana	BSC	TTAGGG	6	Pezizomycotina	yes	CM018003.1;CM018017.1	2/16	30
Alternaria solani	ASL	TTAGGG	6	Pezizomycotina	yes	CP022024.1;CP022025.1;CP022026.1;CP022027.1;CP022028.1;CP022029.1;CP022030.1;CP022031.1;CP022032.1;CP022033.1	10/10	126
Alternaria brassicae	ABR	TAAGGG	6	Pezizomycotina	yes	CM016504.1;CM016505.1;CM016506.1;CM016507.1;CM016508.1;CM016509.1;CM016510.1;CM016511.1;CM016512.1;CM016513.1	10/10	150

Parastagonospora nodorum	PNO	TTAGGG	6	Pezizomycotina	yes	CP022852.1;CP022853.1; CP022855.1;CP022854.1; CP022856.1;CP022858.1; CP022857.1;CP022860.1; CP022859.1;CP022861.1; CP022863.1;CP022862.1; CP022865.1;CP022864.1; CP022868.1;CP022866.1; CP022870.1;CP022873.1; CP022871.1;CP022867.1	22/22	317
Blumeria graminis	BGT	TTAGGG	6	Pezizomycotina	yes	LR026984.1;LR026986.1; LR026988.1;LR026989.1; LR026991.1;LR026992.1; LR026993.1	7/12	421
Botrytis cinerea	BFU	TTAGGG	6	Pezizomycotina	yes	NC_037312.1;NC_037316.1; NC_037322.1;NC_037320.1; NC_037325.1;NC_037311.1; NC_037317.1;NC_037321.1; NC_037324.1;NC_037315.1; NC_037318.1;NC_037310.1; NC_037323.1;NC_037313.1; NC_037319.1;NC_037327.1; NC_037326.1	17/18	129
Sclerotinia sclerotiorum	SSL	TTAGGG	6	Pezizomycotina	yes	CP017828.1;CP017815.1; CP017822.1;CP017818.1; CP017823.1;CP017829.1; CP017827.1;CP017825.1; CP017817.1;CP017821.1; CP017814.1;CP017819.1; CP017820.1;CP017816.1	14/16	195
Pyricularia pennisetigena	PPE	TTAGGG	6	Pezizomycotina	yes	NC_043742.1;NC_043743.1; NC_043744.1	3/5	102
Pyricularia oryzae	POR	TTAGGG	6	Pezizomycotina	yes	CP050920.1;CP050921.1; CP050922.1;CP050923.1; CP050924.1;CP050925.1; CP050926.1;CP050927.1; CP050928.1	9/9	196
Pyricularia grisea	PGR	TTAGGG	6	Pezizomycotina	yes	NC_044975.1	1/3	114
Valsa mali	VMA	TTAGGG	6	Pezizomycotina	yes	CM003098.1;CM003099.1; CM003105.1	3/13	150
Colletotrichum higginsianum	COI	TTAGGG	6	Pezizomycotina	yes	NC_030954.1;NC_030955.1; NC_030956.1;NC_030957.1; NC_030958.1;NC_030959.1	6/6	138
Verticillium dahliae	VDA	TTAGGG	6	Pezizomycotina	yes	CM014044.1	1/8	459
Drechmeria coniospora	DCO	CCGTTGCTGT TG	12	Pezizomycotina	yes	CM004174.1;CM004175.1; CM004176.1	3/3	333

Cordyceps militaris	CMT	TTAGGG	6	Pezizomycotina	yes	CP023328.1;CP023327.1; CP023326.1;CP023322.1; CP023325.1;CP023323.1; CP023324.1	7/7	156
Trichoderma reesei	TRE	TTAGGG	6	Pezizomycotina	yes	CP021297.1;CP021298.1; CP021299.1;CP021300.1; CP021301.1;CP021302.1	6/7	94
Metarhizium brunneum	MBR	TTAGGG	6	Pezizomycotina	yes	CP058932.1;CP058933.1; CP058934.1;CP058935.1; CP058936.1;CP058937.1; CP058938.1	7/8	150
Pochonia chlamydosporia	PCH	TTAGGG	6	Pezizomycotina	yes	NC_035790.1;NC_035793.1; NC_035794.1;NC_035795.1; NC_035796.1	5/7	156
Epichloe festucae	EFE	TTAGGG	6	Pezizomycotina	yes	CP031385.1;CP031386.1; CP031387.1;CP031388.1; CP031389.1;CP031390.1; CP031391.1	7/8	228
Fusarium verticillioides	FVR	/	0	Pezizomycotina	no	/	/	/
Fusarium pseudograminearum	FPU	TTAGGG	6	Pezizomycotina	yes	NC_031951.1;NC_031952.1; NC_031954.1	3/4	102
Fusarium venenatum	FVE	TTAGGG	6	Pezizomycotina	yes	NC_038012.1;NC_038013.1; NC_038014.1;NC_038015.1	4/5	156
Fusarium circinatum	FCI	/	0	Pezizomycotina	no	/	/	/
Fusarium graminearum	FGR	TTAGGG	6	Pezizomycotina	yes	HG970332.2;HG970333.2; HG970334.2;HG970335.2	4/6	144
Fusarium culmorum	FCU	TTAGGG	6	Pezizomycotina	yes	CP064747.1;CP064748.1; CP064749.1;CP064750.1	4/6	276
Fusarium oxysporum	FOX	TTAGGG	6	Pezizomycotina	yes	NC_031000.1;NC_030990.1	2/15	168
Thermothielavioides terrestris	TTT	TTAGGG	6	Pezizomycotina	yes	NC_016457.1;NC_016458.1; NC_016459.1;NC_016460.1; NC_016461.1;NC_016462.1	6/6	315
Thermothelomyces thermophilus	TTH	TTAGGG	6	Pezizomycotina	yes	NC_016472.1;NC_016473.1; NC_016474.1;NC_016475.1; NC_016476.1;NC_016477.1; NC_016478.1	7/7	193
Podospora comata	PCO	/	0	Pezizomycotina	no	/	/	/
Neurospora crassa	NCR	TTAGGG	6	Pezizomycotina	yes	NC_026501.1;NC_026502.1; NC_026503.1;NC_026504.1; NC_026505.1;NC_026506.1; NC_026507.1	7/8	312
Exophiala lecanii-corni	ELC	TTAGGG	6	Pezizomycotina	yes	CP034370.1;CP034371.1; CP034372.1;CP034373.1; CP034374.1;CP034375.1; CP034377.1;CP034378.1; CP034379.1;CP034380.1; CP034381.1;CP034376.1	12/13	92

Talaromyces pinophilus	TPI	TTAGGG	6	Pezizomycotina	yes	CP017344.1;CP017345.1; CP017346.1;CP017347.1; CP017348.1;CP017349.1; CP017350.1;CP017351.1	8/9	141
Talaromyces rugulosus	TRU	TTAGGG	6	Pezizomycotina	yes	NC_049561.1;NC_049562.1; NC_049563.1;NC_049564.1; NC_049565.1;NC_049566.1	6/6	180
Talaromyces marneffei	TMF	TTAGG[G,A]	10	Pezizomycotina	yes	CP015868.1;CP015869.1; CP015871.1;CP015872.1; CP015873.1;CP015875.1	6/8	800
Talaromyces funiculosus	TFU	TTAGGG	6	Pezizomycotina	yes	CP036227.1;CP036228.1; CP036229.1;CP036231.1; CP036232.1	5/21	126
Penicillium polonicum	PPO	TTTAGGG	7	Pezizomycotina	yes	CM024080.1;CM024081.1; CM024082.1	3/4	182
Monascus purpureus	MPU	/	0	Pezizomycotina	no	/	/	/
Aspergillus fumigatus	AFM	TTAGGG	6	Pezizomycotina	yes	NC_007194.1;NC_007195.1; NC_007196.1;NC_007197.1; NC_007198.1;NC_007199.1; NC_007200.1;NC_007201.1	8/8	119
Aspergillus nidulans	ANI	TTAGGG	6	Pezizomycotina	yes	BN001302.1;BN001305.1; BN001306.1;BN001307.1; BN001308.1	5/8	230
Aspergillus sojae	ASO	TTAGGGTCAA CA	12	Pezizomycotina	yes	CP035525.1;CP035526.1; CP035527.1;CP035530.1	4/8	150
Aspergillus parasiticus	APA	TTAGGGTCAA CA	12	Pezizomycotina	yes	CP051028.1;CP051029.1; CP051030.1;CP051032.1; CP051033.1;CP051034.1	6/8	85
Aspergillus oryzae	AOR	TTAGGGTCAA CA	12	Pezizomycotina	yes	NC_036435.1;NC_036438.1; NC_036439.1;NC_036441.1; NC_036442.1	5/9	89
Aspergillus flavus	AFV	TTAGGGTCAA CA	12	Pezizomycotina	yes	CP061804.1;CP061805.1; CP061806.1;CP061807.1; CP061809.1;CP061810.1	6/9	260
Sugiyamaella lignohabitans	SLB	/	0	Saccharomycotina	no	/	/	/
[Candida] hispaniensis	CHI	TTGACGAGAG	10	Saccharomycotina	yes	LS992270.1	1/6	207
[Candida] auris	CAU	TTAGGTGGTG TCTGGGTTTC	20	Saccharomycotina	yes	CP060360.1;CP060361.1; CP060362.1;CP060363.1; CP060364.1;CP060365.1; CP060366.1	6/7	397
[Candida] intermedia	CIN	TTAGGGAGGT AGAGGTTTTT C	21	Saccharomycotina	yes	LT635756.1;LT635757.1; LT635758.1;LT635759.1; LT635760.1;LT635761.1; LT635762.1	7/8	400
Clavispora lusitaniae	CLU	TTAGGGAGGT ACTGATGTTC T	21	Saccharomycotina	yes	CP038484.1;CP038485.1; CP038486.1;CP038487.1; CP038489.1;CP038490.1; CP038491.1;CP038488.1	8/8	396

Metschnikowia aff. pulcherrima	MAP	TTAGGGAGGTACGGGTGTCTTAGCATC	27	Saccharomycotina	yes	CP034456.1;CP034457.1; CP034458.1	3/7	343
Metschnikowia reukaufii	MRE	TTAGGGATGTACTGATTTATC	21	Saccharomycotina	yes	CM010597.1;CM010598.1; CM010604.1	3/9	406
Yarrowia lipolytica	YLI	TTAGTCAGGG	10	Saccharomycotina	yes	CP061012.1;CP061014.1; CP061015.1;CP061016.1; CP061017.1	5/7	663
Saccharomycopsis malanga	SMA	TAAGGGTGTCAGTGGGG	17	Saccharomycotina	yes	CP025321.1;CP025322.1; CP025323.1;CP025324.1; CP025325.1;CP025326.1	6/7	227
Saccharomycopsis fibuligera	SFI	TAAGGGTGGTG	11	Saccharomycotina	yes	CP015978.1;CP015983.1; CP015984.1	3/7	146
Hyphopichia pseudoburtonii	HPS	TACGGGTCTTTTCTACGAGGGTGAGGAGG	29	Saccharomycotina	yes	CP024751.1;CP024752.1; CP024753.1;CP024754.1; CP024755.1;CP024756.1; CP024757.1;CP024758.1	8/8	816
Hyphopichia burtonii	HBU	/	0	Saccharomycotina	no	/	/	/
Candida orthopsilosis	COT	/	0	Saccharomycotina	no	/	/	/
Candida dubliniensis	CDU	TACGGATGTCTAACTTCTGGTG	22	Saccharomycotina	yes	NC_012867.1	1/8	254
Candida tropicalis	CTP	TAAGGATGTCACGATCATTGGTG	23	Saccharomycotina	yes	CP047869.1;CP047870.1; CP047871.1;CP047872.1; CP047873.1;CP047874.1; CP047875.1	7/7	199
Candida albicans	CAL	TAAGGATGTCACGATCATTGGTG	23	Saccharomycotina	yes	CP032017.1	1/8	931
Debaryomyces hansenii	DHA	TAGGGATGTTGAGGTG	16	Saccharomycotina	yes	NC_006044.2;NC_006048.2	2/8	315
Scheffersomyces stipitis	SST	TATGGATCTTTTCACGTCTTGCGG	24	Saccharomycotina	yes	NC_009068.1;NC_009042.1; NC_009043.1;NC_009044.1; NC_009045.1;NC_009046.1; NC_009047.1;NC_009048.1	8/8	558
Millerozyma farinosa	MFA	/	0	Saccharomycotina	no	/	/	/
Saccharomycetaceae sp.	AAC	TATGGGTCTCTCAGCGGTGTGGTG	24	Saccharomycotina	yes	CP006020.1;CP006021.1; CP006022.1;CP006023.1; CP006025.1;CP006026.1	6/8	697
Kazachstania naganishii	KNA	/	0	Saccharomycotina	no	/	/	/
Kazachstania africana	KAF	/	0	Saccharomycotina	no	/	/	/
Tetrapisispora blattae	TBL	/	0	Saccharomycotina	no	/	/	/
Tetrapisispora phaffii	TPF	/	0	Saccharomycotina	no	/	/	/
Zygotorulaspora mrakii	ZMR	TAGGGGTGCGGTG	13	Saccharomycotina	yes	NC_050719.1;NC_050720.1; NC_050722.1;NC_050723.1; NC_050724.1;NC_050726.1	6/8	355

Eremothecium cymbalariae	ERC	TACGGGTCTC TCAGCGGTGT GGTG	24	Saccharomycotina	yes	NC_016451.1;NC_016452.1; NC_016453.1;NC_016454.1; NC_016456.1	5/8	298
Eremothecium gossypii	AGO	TACGGGTCTC TCAGCGGTGT GGTG	24	Saccharomycotina	yes	NC_005782.2;NC_005783.5; NC_005784.3;NC_005785.6; NC_005786.2;NC_005787.5; NC_005788.4	7/8	672
Naumovozyma dairenensis	NDI	/	0	Saccharomycotina	no	/	/	/
Naumovozyma castellii	NCS	/	0	Saccharomycotina	no	/	/	/
[Candida] glabrata	CGR	TCTGGGTGCT GTGGGG	16	Saccharomycotina	yes	CP048234.1;CP048230.1; CP048240.1;CP048241.1; CP048236.1;CP048235.1; CP048232.1;CP048238.1; CP048233.1;CP048237.1; CP048231.1;CP048239.1; CP048242.1	13/13	817
Zygosaccharomyces parabailii	ZPA	/	0	Saccharomycotina	no	/	/	/
Zygosaccharomyces rouxii	ZRO	/	0	Saccharomycotina	no	/	/	/
Torulaspora delbrueckii	TDL	TAAGGTTGTG GTG	13	Saccharomycotina	yes	NC_016501.1	1/8	214
Lachancea mirantina	LMI	TGGAGGAGGA GTG	13	Saccharomycotina	yes	LT598466.1;LT598469.1	2/8	52
Lachancea dasiensis	LDA	/	0	Saccharomycotina	no	/	/	/
Lachancea nothofagi	LNO	/	0	Saccharomycotina	no	/	/	/
Lachancea thermotolerans	LTH	GTGGAGTAC	9	Saccharomycotina	yes	NC_013077.1;NC_013082.1; NC_013084.1	3/8	153
Lachancea meyersii	LME	/	0	Saccharomycotina	no	/	/	/
Lachancea fermentati	LFE	/	0	Saccharomycotina	no	/	/	/
Lachancea kluyveri	LKL	TGGACATGCG TACTGTGAGG TCTGGG	26	Saccharomycotina	yes	CM000687.1;CM000688.1; CM000689.1;CM000691.1	4/8	648
Saccharomyces eubayanus	SEU	T(G)2-3(TG)1-6	5~16	Saccharomycotina	/	/	/	/
Saccharomyces arboricola	SAR	T(G)2-3(TG)1-6	5~16	Saccharomycotina	/	/	/	/
Saccharomyces boulardii	SBO	T(G)2-3(TG)1-6	5~16	Saccharomycotina	/	/	/	/
Saccharomyces kudriavzevii	SKU	T(G)2-3(TG)1-6	5~16	Saccharomycotina	/	/	/	/
Saccharomyces pastorianus	SPS	T(G)2-3(TG)1-6	5~16	Saccharomycotina	/	/	/	/
Saccharomyces paradoxus	SPR	T(G)2-3(TG)1-6	5~16	Saccharomycotina	/	/	/	/
Saccharomyces cerevisiae	SCE	T(G)2-3(TG)1-6	5~16	Saccharomycotina	/	/	/	/
Kluyveromyces lactis	KLA	TTAGGTATGT GGTGTACGGA TTTGA	25	Saccharomycotina	yes	NC_006037.1;NC_006038.1; NC_006039.1;NC_006040.1; NC_006041.1;NC_006042.1	6/8	708
Kluyveromyces marxianus	KMX	TTAGGTATGT GGTGTACGGA TTTGA	25	Saccharomycotina	yes	NC_036030.1;NC_036025.1; NC_036028.1;NC_036029.1; NC_036032.1;NC_036027.1; NC_036031.1;NC_036026.1	8/9	750
Ogataea parapolymorpha	OPA	GGTGGCGG	8	Saccharomycotina	yes	NC_027860.1;NC_027863.1	2/7	78
Pichia kudriavzevii	PKZ	GTGTGTTACA ATATGAACTA GGAGCGAG	28	Saccharomycotina	yes	NC_042507.1;NC_042509.1; NC_042510.1	3/5	529

Komagataella phaffii	PPA	ATGCTGG	7	Saccharomycotina	yes	LT962476.1;LT962477.1; LT962478.1;LT962479.1	4/4	237
Komagataella pastoris	KPA	ATGCTGG	7	Saccharomycotina	yes	CP014584.1;CP014585.1; CP014587.1;CP014588.1; CP014593.1;CP014586.1	6/11	357
Cyberlindnera jadinii	CJA	TCTGGGTG	8	Saccharomycotina	yes	DG000065.1;DG000066.1; DG000067.1;DG000069.1; DG000070.1	5/13	443
Schizosaccharomyces pombe	SPO	G2–8TTAC(A)	0	Taphrinomycotina	no	/	/	/
Pyricularia sp.	PYR	TTAGGG	6	Pezizomycotina	yes	RRCJ01000018.1; RRCJ01000016.1; RRCJ01000013.1 ;RRCJ01000043.1; RRCJ01000044.1; RRCJ01000047.1; RRCJ01000004.1; RRCJ01000007.1; RRCJ01000011.1; RRCJ01000015.1; RRCJ01000020.1; RRCJ01000025.1; RRCJ01000031.1	13/49	102
Byssochlamys spectabilis	BSP	TTTAGGG	7	Pezizomycotina	yes	CM012214.1;CM012215.1; CM012216.1;CM012217.1; CM012218.1;CM012219.1; CM012220.1;CM012221.1	8/10	112
Exserohilum turcica	ETE	TTAGGG	6	Pezizomycotina	yes	CP054649.1;CP054643.1; CP054652.1;CP054650.1; CP054640.1;CP054627.1; CP054628.1;CP054630.1; CP054636.1;CP054654.1; CP054635.1;CP054638.1; CP054633.1;CP054642.1; CP054653.1;CP054641.1; CP054647.1;CP054639.1; CP054632.1;CP054644.1; CP054629.1;CP054637.1; CP054646.1;CP054634.1	24/30	181
Antonospora locustae	ALO	/	0	Fungi incertae sedis	no	/	/	/
Encephalitozoon romaleae	ERO	/	0	Fungi incertae sedis	no	/	/	/
Encephalitozoon intestinalis	EIN	/	0	Fungi incertae sedis	no	/	/	/
Encephalitozoon hellem	EHE	/	0	Fungi incertae sedis	no	/	/	/
Encephalitozoon cuniculi	ECU	GGTTATGTGT	10	Fungi incertae sedis	yes	NC_003242.2	1/11	70
Pleurotus ostreatus	POS	TTAGGG	6	Agaricomycotina;Basid iomycota	yes	CM016808.1;CM016811.1; CM016812.1;CM016813.1; CM016814.1;CM016816.1; CM016817.1	7/11	48

Pyrrhoderma noxium	PNX	TGTTAGG	7	Agaricomycotina;Basidiomycota	yes	CM008251.1;CM008252.1;CM008254.1;CM008255.1;CM008256.1;CM008258.1;CM008260.1	7/13	463
Ustilago maydis	UMA	TTAGGG	6	Ustilaginomycotina;Basidiomycota	yes	NC_026481.1;NC_026497.1	2/23	372
Ustilago bromivora	UBR	TTAGGG	6	Ustilaginomycotina;Basidiomycota	yes	LT558129.1;LT558126.1;LT558118.1;LT558125.1;LT558134.1;LT558120.1;LT558121.1;LT558127.1;LT558137.1;LT558122.1;LT558135.1;LT558128.1;LT558138.1;LT558124.1;LT558133.1;LT558136.1;LT558130.1;LT558123.1;LT558119.1;LT558117.1;LT558139.1;LT558131.1	22/24	208
Trametes hirsuta	THI	TTAGGG	6	Agaricomycotina;Basidiomycota	yes	CP019373.1;CP019382.1;CP019376.1;CP019377.1;CP019381.1;CP019378.1;CP019371.1;CP019372.1;CP019380.1;CP019379.1;CP019375.1;CP019370.1;CP019374.1	13/13	286
Sporisorium reilianum	SRE	/	0	Ustilaginomycotina;Basidiomycota	no	/	/	/
Sporisorium scitamineum	SSC	TTAGGG	6	Ustilaginomycotina;Basidiomycota	yes	CP010923.1;CP010916.1;CP010927.1;CP010913.1;CP010919.1;CP010928.1;CP010917.1;CP010925.1;CP010924.1;CP010915.1;CP010934.1;CP010935.1;CP010914.1;CP010931.1;CP010926.1;CP010930.1;CP010929.1;CP010938.1;CP010932.1;CP010936.1;CP010918.1;CP010922.1;CP010921.1;CP010920.1	24/27	139
Sporisorium graminicola	SGR	TTAGGG	6	Ustilaginomycotina;Basidiomycota	yes	NC_043737.1;NC_043735.1;NC_043726.1;NC_043738.1;NC_043727.1;NC_043732.1;NC_043719.1;NC_043721.1;NC_043736.1;NC_043734.1;NC_043723.1;NC_043722.1;NC_043731.1;NC_043730.1;NC_043725.1;NC_043729.1;NC_043728.1;NC_043733.1;NC_043737.1;NC_043720.1;NC_043724.1;NC_043739.1	22/22	328

Malassezia restricta	MRT	TTAGTG	6	Ustilaginomycotina;Basidiomycota	yes	NC_040194.1;NC_040193.1;NC_040195.1;NC_040194.1;NC_040198.1;NC_040196.1;NC_040200.1;NC_040199.1;NC_040201.1;NC_040197.1	10/10	312
Malassezia globosa	MGL	TTAGTG	6	Ustilaginomycotina;Basidiomycota	yes	CP046436.1;CP046435.1;CP046439.1;CP046433.1;CP046431.1;CP046432.1;CP046437.1;CP046436.1;CP046434.1;CP046438.1;CP046440.1	10/11	209
Malassezia sympodialis	MSYM	TTAAGTG	7	Ustilaginomycotina;Basidiomycota	yes	LT671791.1;LT671792.1;LT671795.1;LT671794.1;LT671789.1;LT671790.1;LT671793.1;LT671796.1	8/8	285
Malassezia furfur	MFU	TTAGGA	6	Ustilaginomycotina;Basidiomycota	yes	CP046237.1;CP046234.1;CP046239.1;CP046236.1;CP046238.1;CP046235.1;CP046240.1;CP046237.1	8/8	431
Hericium erinaceus	HER	TTGGA	5	Agaricomycotina;Basidiomycota	yes	CM021160.1;CM021163.1;CM021166.1;CM021167.1;CM021169.1;CM021170.1;CM021172.1;CM021173.1	8/15	65
Flammulina velutipes	FVL	TTAGGG	6	Agaricomycotina;Basidiomycota	yes	CM022370.1;CM022377.1;CM022376.1;CM022373.1;CM022375.1;CM022372.1;CM022374.1;CM022378.1;CM022371.1;CM022369.1;CM022370.1	11/11	286
Cryptococcus wingfieldii	CWI	TTAGGGGG	8	Agaricomycotina;Basidiomycota	no	/	/	/
Cryptococcus neoformans	CNV	TTAGGGGG	8	Agaricomycotina;Basidiomycota	yes	NC_006679.1;NC_006684.1;NC_006685.1;NC_006694.1;NC_006693.1;NC_006680.1;NC_006692.1;NC_006686.1;NC_006687.1;NC_006670.1;NC_006683.1;NC_006681.1;NC_006682.1;NC_006691.1	14/14	96
Cryptococcus gattii	CGI	TTAGGGGG	8	Agaricomycotina;Basidiomycota	yes	NC_014939.1;NC_014947.1;NC_014949.1;NC_014940.1;NC_014946.1;NC_014950.1;NC_014948.1;NC_014944.1;NC_014951.1;NC_014938.1;NC_014942.1	11/14	104
Cryptococcus floricola	CFL	TTAGCGGGG	9	Agaricomycotina;Basidiomycota	yes	CM016930.1;CM016932.1;CM016940.1;CM016939.1;CM016942.1;CM016934.1;CM016931.1;CM016933.1;CM016938.1;CM016936.1	10/14	91

Cryptococcus cf. gattii	CGA	TTAG(3-4)	7	Agaricomycotina;Basidiomycota	yes	CM018872.1;CM018860.1;CM018868.1;CM018865.1;CM018864.1;CM018861.1;CM018867.1;CM018870.1;CM018866.1;CM018869.1;CM018871.1;CM018862.1;CM018863.1	13/13	79
Apiotrichum mycotoxinovorans	AMY	TTAGGGG	7	Agaricomycotina;Basidiomycota	yes	CP053620.1;CP053624.1	2/7	97
Agaricus bisporus	ABP	TTAGGGGG	8	Agaricomycotina;Basidiomycota	yes	CP015481.1;CP015476.1;CP015482.1;CP015479.1;CP015472.1;CP015471.1;CP015470.1;CP015473.1;CP015474.1;CP015477.1	10/13	88
Hypsizygus marmoreus	HMA	TTAGGG	6	Agaricomycotina;Basidiomycota	yes	CM024087.1;CM024086.1;CM024085.1;CM024093.1;CM024095.1;CM024090.1;CM024092.1;CM024091.1;CM024088.1;CM024094.1;CM024089.1;CM024084.1	12/12	145

Table S3A: SV frequency of telomere (HSA)

end	chr_name	chr	telomere length	motif count	SV count	SV frequency
left	NC_060925.1	chr1	2700	438	98	0.22
left	NC_060926.1	chr2	3616	570	175	0.31
left	NC_060927.1	chr3	2636	436	70	0.16
left	NC_060928.1	chr4	3258	533	120	0.23
left	NC_060929.1	chr5	2295	367	53	0.14
left	NC_060930.1	chr6	2892	475	36	0.08
left	NC_060931.1	chr7	3412	554	93	0.17
left	NC_060932.1	chr8	2512	405	104	0.26
left	NC_060933.1	chr9	3630	585	119	0.20
left	NC_060934.1	chr10	2636	429	68	0.16
left	NC_060935.1	chr11	1983	319	35	0.11
left	NC_060936.1	chr12	3101	487	124	0.25
left	NC_060937.1	chr13	2541	423	7	0.02
left	NC_060938.1	chr14	2073	344	4	0.01
left	NC_060939.1	chr15	3253	531	161	0.30
left	NC_060940.1	chr16	2268	374	71	0.19
left	NC_060941.1	chr17	2205	364	76	0.21
left	NC_060942.1	chr18	2942	336	20	0.06
left	NC_060943.1	chr19	2281	357	68	0.19
left	NC_060944.1	chr20	2723	443	66	0.15
left	NC_060945.1	chr21	3008	460	53	0.12
left	NC_060946.1	chr22	4574	736	74	0.10
left	NC_060947.1	chrX	1822	302	37	0.12
left	NC_060948.1	chrY	5654	941	34	0.04
right	NC_060925.1	chr1	3185	512	102	0.20
right	NC_060926.1	chr2	2621	424	64	0.15
right	NC_060927.1	chr3	4612	746	172	0.23
right	NC_060928.1	chr4	2296	373	45	0.12
right	NC_060929.1	chr5	1553	255	42	0.16
right	NC_060930.1	chr6	2778	450	52	0.12
right	NC_060931.1	chr7	2219	357	70	0.20

right	NC_060932.1	chr8	1842	454	152	0.33
right	NC_060933.1	chr9	2970	474	153	0.32
right	NC_060934.1	chr10	3196	515	109	0.21
right	NC_060935.1	chr11	2589	422	44	0.10
right	NC_060936.1	chr12	2337	375	40	0.11
right	NC_060937.1	chr13	3499	572	61	0.11
right	NC_060938.1	chr14	1652	267	34	0.13
right	NC_060939.1	chr15	2929	464	61	0.13
right	NC_060940.1	chr16	2678	435	49	0.11
right	NC_060941.1	chr17	2996	476	52	0.11
right	NC_060942.1	chr18	3489	557	132	0.24
right	NC_060943.1	chr19	2934	465	57	0.12
right	NC_060944.1	chr20	3153	490	118	0.24
right	NC_060945.1	chr21	4548	730	79	0.11
right	NC_060946.1	chr22	2931	467	87	0.19
right	NC_060947.1	chrX	2943	468	101	0.22
right	NC_060948.1	chrY	6389	1023	113	0.11

Table S3B: SV frequency of telomere (ATH)

end	chr_name	chr	telomere length	motif count	SV count	SV frequency
left	CP087126.1	chr1	3257	439	9	0.02
left	CP087127.1	chr2	2946	379	4	0.01
left	CP087128.1	chr3	3263	443	35	0.08
left	CP087129.1	chr4	0	0	0	NAN
left	CP087130.1	chr5	3274	460	12	0.03
right	CP087126.1	chr1	3562	508	2	0.00
right	CP087127.1	chr2	3611	497	84	0.17
right	CP087128.1	chr3	3006	424	11	0.03
right	CP087129.1	chr4	3385	467	64	0.14
right	CP087130.1	chr5	4480	475	30	0.06

Table S3C: SV frequency of telomere (YLI)

end	chr_name	chr	telomere length	motif count	SV count	SV frequency
left	CP061012.1	chrA	369	37	0	0.00
left	CP061013.1	chrB	0	0	0	NAN
left	CP061014.1	chrC	663	63	13	0.21
left	CP061015.1	chrD	256	21	4	0.19
left	CP061016.1	chrE	0	0	0	NAN
left	CP061017.1	chrF	0	0	0	NAN
right	CP061012.1	chrA	520	48	0	0.00
right	CP061013.1	chrB	0	0	0	NAN
right	CP061014.1	chrC	478	38	13	0.34
right	CP061015.1	chrD	434	42	6	0.14
right	CP061016.1	chrE	303	30	1	0.03
right	CP061017.1	chrF	383	38	1	0.03

Table S4A: coefficient for kmer length and repeat times in scoring and its detection result in five end of ATH

kmer length	repeat times	kmer	suppChr
1	1	CCCTAAA	4
0.1	1	CCCTAAA	4
0.2	1	CCCTAAA	4
0.3	1	CCCTAAA	4
0.4	1	CCCTAAA	4
0.5	1	CCCTAAA	4
0.6	1	CCCTAAA	4
0.7	1	CCCTAAA	4
0.8	1	CCCTAAA	4
0.9	1	CCCTAAA	4
1	0.1	CCCTAAA	4
1	0.2	CCCTAAA	4
1	0.3	CCCTAAA	4
1	0.4	CCCTAAA	4
1	0.5	CCCTAAA	4
1	0.6	CCCTAAA	4
1	0.7	CCCTAAA	4
1	0.8	CCCTAAA	4
1	0.9	CCCTAAA	4
0.1	0.9	CCCTAAA	4
0.2	0.8	CCCTAAA	4
0.3	0.7	CCCTAAA	4
0.4	0.6	CCCTAAA	4
0.5	0.5	CCCTAAA	4
0.6	0.4	CCCTAAA	4
0.7	0.3	CCCTAAA	4
0.8	0.2	CCCTAAA	4
0.9	0.1	CCCTAAA	4

Table S4B: coefficient for kmer length and repeat times in scoring and its detection result in five end of YLI

kmer length	repeat times	kmer	suppChr
1	1	ACTAACCCTG	3
0.1	1	ACTAACCCTG	3
0.2	1	ACTAACCCTG	3
0.3	1	ACTAACCCTG	3
0.4	1	ACTAACCCTG	3
0.5	1	ACTAACCCTG	3
0.6	1	ACTAACCCTG	3
0.7	1	ACTAACCCTG	3
0.8	1	ACTAACCCTG	3
0.9	1	ACTAACCCTG	3
1	0.1	ACTAACCCTG	1
1	0.2	ACTAACCCTG	2
1	0.3	ACTAACCCTG	2
1	0.4	ACTAACCCTG	3
1	0.5	ACTAACCCTG	3
1	0.6	ACTAACCCTG	3
1	0.7	ACTAACCCTG	3
1	0.8	ACTAACCCTG	3
1	0.9	ACTAACCCTG	3
0.1	0.9	ACTAACCCTG	3
0.2	0.8	ACTAACCCTG	3
0.3	0.7	ACTAACCCTG	3
0.4	0.6	ACTAACCCTG	3
0.5	0.5	ACTAACCCTG	3
0.6	0.4	ACTAACCCTG	3
0.7	0.3	ACTAACCCTG	2
0.8	0.2	ACTAACCCTG	2
0.9	0.1	ACTAACCCTG	1

Table S5: the possible reason for detection failure by TelFinder

count	**species**	**reason**
8	*Saccharomyces eubayanus, Saccharomyces arboricola,Saccharomyces boulardii, Saccharomyces kudriavzevii, Saccharomyces pastorianus, Saccharomyces paradoxus, Saccharomyces paradoxus, Schizosaccharomyces pombe*	Variable telomeric repeat sequence
27	*Cercospora sojina, Cercospora beticola, Fusarium verticillioides, Fusarium circinatum, Podospora comata, Podospora comata, Podospora comata, Hyphopichia burtonii, Candida orthopsilosis, Millerozyma farinosa, Kazachstania naganishii, Kazachstania africana, Tetrapisispora blattae, Tetrapisispora phaffii, Naumovozyma dairenensis, Naumovozyma castellii, Zygosaccharomyces parabailii, Zygosaccharomyces rouxii, Lachancea dasiensis, Lachancea nothofagi, Lachancea meyersii, Lachancea fermentati, Lachancea fermentati, Encephalitozoon romaleae, Encephalitozoon intestinalis, Encephalitozoon intestinalis, Sporisorium reilianum*	Too few repeat times to distinguish
