# Supplementary material for: Correction for Sun et al., “Large-Scale Detection of Telomeric Motif Sequences in Genomic Data Using TelFinder”
Source: Microbiol Spectr. 2023 Oct 13;11(6):e02777-23. doi: 10.1128/spectrum.02777-23 (PMC10715131; doi:10.1128/spectrum.02777-23)
Supplement: Supplemental material — for published article; Table S1 is revised [file spectrum.02777-23-s0001.docx]

Table S1: Application of TelFinder to a reference dataset in Telomerase Database

|  | **species** | **Sequences** | **References** | **genomic** | **TelFinder** | **Accordance** | **chr with telomere** | **telchrN/allchrN** | **max Tellen** |
| --- | --- | --- | --- | --- | --- | --- | --- | --- | --- |
| **Invertebrates** | Tribolium castaneum (red flour beetle) | TCAGG | Osanai, M., Kojima, K. K., Futahashi, R., Yaguchi, S., & Fujiwara, H. (2006). Identification and characterization of the telomerase reverse transcriptase of Bombyx mori (silkworm) and Tribolium castaneum (flour beetle). Gene, 376(2), 281–289. | yes | TCAGG | yes | NC_007417.3; NC_007424.3; NC_007425.3 | 3/11 | 199 |
|  | Tribolium confusum | TCAGG | Mravinac, B., Meštrović, N., Cavrak, V. V., & Plohl, M. (2011). TCAGG, an alternative telomeric sequence in insects. Chromosoma, 120(4), 367–376. | yes | TCAGG | yes | CM032790.1 | 1/8 | 373 |
|  | Apis mellifera (honey bee) | TTAGG | Sahara, K., Marec, F., & Traut, W. (1999). TTAGG telomeric repeats in chromosomes of some insects and other arthropods. Chromosome research : an international journal on the molecular, supramolecular and evolutionary aspects of chromosome biology, 7(6), 449–460. | yes | TTAGG | yes | NC_037644.1; NC_037640.1; NC_037645.1; NC_037638.1; NC_037639.1; NC_037651.1; NC_037647.1; NC_037653.1; NC_037641.1; NC_037650.1; NC_037643.1; NC_037652.1; NC_037649.1 | 13/17 | 11785 |
|  | Mamestra brassicae (cabbage moth) | TTAGG | Frydrychová, R., Grossmann, P., Trubac, P., Vítková, M., & Marec, F. (2004). Phylogenetic distribution of TTAGG telomeric repeats in insects. Genome, 47(1), 163–178. | yes | TTAGG | yes | LR990988.1; LR990991.1; LR990992.1; LR990993.1; LR990995.1; LR990996.1; LR990997.1; LR990998.1; LR990999.1; LR991000.1; LR991001.1; LR991003.1; LR991004.1; LR991005.1; LR991006.1; LR991007.1; LR991008.1; LR991009.1; LR991010.1; LR991011.1; LR991012.1; LR991013.1; LR991014.1; LR991015.1; LR991016.1; LR991017.1; LR990987.1 | 23/32 | 15850 |
|  | Ascaris suum | TTAGGC | Teixeira, M. T., & Gilson, E. (2005). Telomere maintenance, function and evolution: the yeast paradigm. Chromosome research : an international journal on the molecular, supramolecular and evolutionary aspects of chromosome biology, 13(5), 535–548. | yes | TTAGGC | yes | CM024168.1; CM024170.1; CM024188.1; CM024172.1; CM024174.1; CM024190.1 | 6/25 | 49704 |
|  | Caenorhabditis elegans | TTAGGC | Cangiano, G., & La Volpe, A. (1993). Repetitive DNA sequences located in the terminal portion of the Caenorhabditis elegans chromosomes. Nucleic acids research, 21(5), 1133–1139. | yes | TTAGGC | yes | NC_003279.8; NC_003280.10; NC_003281.10; NC_003282.8; NC_003283.11; NC_003284.9 | 6/7 | 845 |
| **Fungi** | Schizosaccharomyces pombe (fission yeast) | G2–8TTAC(A) | Joseph, I., & Lustig, A. J. (2007). Telomeres in meiotic recombination: the yeast side story. Cellular and molecular life sciences : CMLS, 64(2), 125–130. | yes | G2–8TTAC(A) | / | / | / | / |
|  | Saccharomyces cerevisiae (baker's yeast) | T(G)2-3(TG)1-6 | McEachern, M. J., & Blackburn, E. H. (1994). A conserved sequence motif within the exceptionally diverse telomeric sequences of budding yeasts. Proceedings of the National Academy of Sciences of the United States of America, 91(8), 3453–3457. | yes | T(G)2-3(TG)1-6 | / | / | / | / |
|  | Saccharomyces bayanus | T(G)2-3(TG)1-6 | Teixeira, M. T., & Gilson, E. (2005). Telomere maintenance, function and evolution: the yeast paradigm. Chromosome research : an international journal on the molecular, supramolecular and evolutionary aspects of chromosome biology, 13(5), 535–548. | yes | T(G)2-3(TG)1-6 | / | / | / | / |
|  | Saccharomyces paradoxus | T(G)2-3(TG)1-6 | Teixeira, M. T., & Gilson, E. (2005). Telomere maintenance, function and evolution: the yeast paradigm. Chromosome research : an international journal on the molecular, supramolecular and evolutionary aspects of chromosome biology, 13(5), 535–548. | yes | T(G)2-3(TG)1-6 | / | / | / | / |
|  | Saccharomyces castellii | TCTGGG(TG)1-4 | Cohn, M., & Blackburn, E. H. (1995). Telomerase in yeast. Science (New York, N.Y.), 269(5222), 396–400. | yes | T(G)2-3(TG)1-6 | / | / | / | / |
|  | Saccharomyces kluyveri | GGGTGGACATGCGTACTGTGAGGTCT | Cohn, M., McEachern, M. J., & Blackburn, E. H. (1998). Telomeric sequence diversity within the genus Saccharomyces. Current genetics, 33(2), 83–91. | yes | TCTGGGTGGACATGCGTACTGTGAGG | yes | CM000687.1; CM000688.1; CM000689.1; CM000691.1 | 4/8 | 598 |
|  | Kluyveromyces lactis | ACGGATTTGATTAGGTATGTGGTGT | McEachern, M. J., & Blackburn, E. H. (1994). A conserved sequence motif within the exceptionally diverse telomeric sequences of budding yeasts. Proceedings of the National Academy of Sciences of the United States of America, 91(8), 3453–3457. | yes | TTAGGTATGTGGTGTACGGATTTGA | yes | NC_006037.1; NC_006038.1; NC_006039.1; NC_006040.1; NC_006041.1; NC_006042.1 | 6/7 | 708 |
|  | Candida albicans | ACGGATGTCTAACTTCTTGGTGT | McEachern, M. J., & Blackburn, E. H. (1994). A conserved sequence motif within the exceptionally diverse telomeric sequences of budding yeasts. Proceedings of the National Academy of Sciences of the United States of America, 91(8), 3453–3457. | yes | TGTACGGATGTCTAACTTCTTGG | yes | CP032017.1 | 1/8 | 931 |
|  | Candida glabrata | CTGGGTGCTGTGGGGT | McEachern, M. J., & Blackburn, E. H. (1994). A conserved sequence motif within the exceptionally diverse telomeric sequences of budding yeasts. Proceedings of the National Academy of Sciences of the United States of America, 91(8), 3453–3457. | yes | TCTGGGTGCTGTGGGG | yes | CP048234.1; CP048230.1; CP048240.1; CP048241.1; CP048236.1; CP048235.1; CP048232.1; CP048238.1; CP048233.1; CP048237.1; CP048231.1; CP048239.1; CP048242.1 | 13/13 | 817 |
|  | Candida orthopsilosis | GGTTAGGATGTAGACAATACTGC | Gunisova, S. (2009). Identification and comparative analysis of telomerase RNAs from Candida species reveal conservation of functional elements. RNA (New York, N.Y.), 15(4), 546–559. | yes | / | / | / | / | / |
|  | Candida pseudotropicalis | ACGGATTTGATTAGTTATGTGGTGT | McEachern, M. J., & Blackburn, E. H. (1994). A conserved sequence motif within the exceptionally diverse telomeric sequences of budding yeasts. Proceedings of the National Academy of Sciences of the United States of America, 91(8), 3453–3457. | yes | TACGGATTTGATTAGTTATGTGGTG | yes | NC_036025.1; NC_036026.1; NC_036027.1; NC_036028.1; NC_036029.1; NC_036030.1; NC_036031.1; NC_036032.1 | 8/9 | 750 |
|  | Candida tropicalis | A[C/A]GGATGTCACGATCATTGGTGT; AAGGATGTCACGATCATTGGTGT | Gunisova, S. (2009). Identification and comparative analysis of telomerase RNAs from Candida species reveal conservation of functional elements. RNA (New York, N.Y.), 15(4), 546–559. McEachern, M. J., & Blackburn, E. H. (1994). A conserved sequence motif within the exceptionally diverse telomeric sequences of budding yeasts. Proceedings of the National Academy of Sciences of the United States of America, 91(8), 3453–3457. | yes | TAAGGATGTCACGATCATTGGTG | yes | CP047875.1; CP047869.1; CP047870.1; CP047871.1; CP047872.1; CP047873.1; CP047874.1 | 7/7 | 199 |
|  | Debaryomyces hansenii | ATGTTGAGGTGTAGGG | Lépingle, A., Casaregola, S., Neuvéglise, C., Bon, E., Nguyen, H., Artiguenave, F., Wincker, P., & Gaillardin, C. (2000). Genomic exploration of the hemiascomycetous yeasts: 14. Debaryomyces hansenii var. hansenii. FEBS letters, 487(1), 82–86. | yes | TAGGGATGTTGAGGTG | yes | NC_006044.2; NC_006048.2 | 2/8 | 315 |
|  | Ashbya gossypii (Eremothecium gossypii) | GTGTGGTGTATGGGTCTCTCAGCG | Dietrich, F. S., Voegeli, S. (2004). The Ashbya gossypii genome as a tool for mapping the ancient Saccharomyces cerevisiae genome. Science (New York, N.Y.), 304(5668), 304–307. | yes | TATGGGTCTCTCAGCGGTGTGGTG | yes | NC_005782.2; NC_005783.5; NC_005784.3; NC_005785.6; NC_005786.2; NC_005787.5; NC_005788.4 | 7/8 | 672 |
|  | Pichia stipitis | GGATCTTTTCACGTCTTGCGGTA | Jeffries, T. W., Grigoriev, I. V. (2007). Genome sequence of the lignocellulose-bioconverting and xylose-fermenting yeast Pichia stipitis. Nature biotechnology, 25(3), 319–326. | yes | GGATCTTTTCACGTCTTGCGGTAT | yes | NC_009068.1; NC_009042.1; NC_009043.1; NC_009044.1; NC_009045.1; NC_009046.1; NC_009047.1; NC_009048.1 | 8/8 | 558 |
|  | Yarrowia lipolytica | GGACGATTG | Teixeira, M. T., & Gilson, E. (2005). Telomere maintenance, function and evolution: the yeast paradigm. Chromosome research : an international journal on the molecular, supramolecular and evolutionary aspects of chromosome biology, 13(5), 535–548. | yes | TCAGGGTTAG | no | CP061012.1; CP061014.1; CP061015.1; CP061016.1; CP061017.1 | 5/7 | 663 |
|  | Clavispora lusitaniae | TCTTTAGGGAGGTACTGATGT | Gunisova, S. (2009). Identification and comparative analysis of telomerase RNAs from Candida species reveal conservation of functional elements. RNA (New York, N.Y.), 15(4), 546–559. | yes | TTTAGGGAGGTACTGATGTTC | yes | CP038484.1; CP038485.1; CP038486.1; CP038487.1; CP038490.1 | 5/8 | 396 |
|  | Aspergillus fumigatus | TTAGGG | Nierman, W. C. (2005). Genomic sequence of the pathogenic and allergenic filamentous fungus Aspergillus fumigatus. Nature, 438(7071), 1151–1156. | yes | TTAGGG | yes | NC_007194.1; NC_007195.1; NC_007196.1; NC_007197.1; NC_007198.1; NC_007199.1; NC_007200.1; NC_007201.1 | 8/8 | 112 |
|  | Aspergillus oryzae | TTAGGGTCAACA | Kusumoto, K. I., Suzuki, S., & Kashiwagi, Y. (2003). Telomeric repeat sequence of Aspergillus oryzae consists of dodeca-nucleotides. Applied microbiology and biotechnology, 61(3), 247–251. | yes | TTAGGGTCAACA | yes | NC_036435.1; NC_036439.1; NC_036441.1; NC_036438.1; NC_036442.1 | 5/9 | 98 |
|  | Aspergillus nidulans (Emericella nidulans) | TTAGGG | Bhattacharyya, A., & Blackburn, E. H. (1997). Aspergillus nidulans maintains short telomeres throughout development. Nucleic acids research, 25(7), 1426–1431. | yes | TTAGGG | yes | BN001302.1; BN001306.1; BN001307.1; BN001305.1; BN001308.1 | 5/8 | 157 |
|  | Histoplasma capsulatum | TTAGGG | Woods, J. P., & Goldman, W. E. (1992). In vivo generation of linear plasmids with addition of telomeric sequences by Histoplasma capsulatum. Molecular microbiology, 6(23), 3603–3610. | yes | TTAGGG | yes | CP069114.1; CP069109.1; CP069110.1; CP069111.1; CP069116.1; CP069112.1 | 6/8 | 198 |
|  | Magnaporthe grisea (rice blast fungus) | TTAGGG | Teixeira, M. T., & Gilson, E. (2005). Telomere maintenance, function and evolution: the yeast paradigm. Chromosome research : an international journal on the molecular, supramolecular and evolutionary aspects of chromosome biology, 13(5), 535–548. | yes | TTAGGG | yes | NC_044975.1 | 1/3 | 105 |
|  | Podospora anserina | TTAGGG | Javerzat, J. P., Bhattacherjee, V., & Barreau, C. (1993). Isolation of telomeric DNA from the filamentous fungus Podospora anserina and construction of a self-replicating linear plasmid showing high transformation frequency. Nucleic acids research, 21(3), 497–504. | yes | TTAGGG | yes | CM030332.1; CM030333.1; CM030335.1; CM030337.1 | 4/8 | 120 |
|  | Neurospora crassa | TTAGGG | Schechtman M. G. (1990). Characterization of telomere DNA from Neurospora crassa. Gene, 88(2), 159–165. | yes | TTAGGG | yes | NC_026502.1; NC_026503.1; NC_026504.1; NC_026505.1; NC_026506.1; NC_026507.1; NC_026501.1 | 7/8 | 309 |
|  | Cryptococcus neoformans (Filobasidiella neoformans) | TTA(G)4-6 | Edman J. C. (1992). Isolation of telomerelike sequences from Cryptococcus neoformans and their use in high-efficiency transformation. Molecular and cellular biology, 12(6), 2777–2783. | yes | TTAGGGGG | yes | NC_006684.1; NC_006686.1; NC_006691.1; NC_006692.1; NC_006693.1; NC_006694.1; NC_006679.1; NC_006680.1; NC_006681.1; NC_006682.1; NC_006683.1; NC_006670.1; NC_006685.1; NC_006687.1 | 14/14 | 93 |
|  | Encephalitozoon cuniculi | G[A/G]GCCT[C/T]CT; GAGCCTTGTTT; GAGACGCAGTGTTGCCAGGATG | Peyret, P., Katinka, M. D. (2001). Sequence and analysis of chromosome I of the amitochondriate intracellular parasite Encephalitozoon cuniculi (Microspora). Genome research, 11(2), 198–207. | yes | GGTTATGTGT | no | NC_003242.2 | 1/11 | 70 |
| **Amoeba** | Dictyostelium discoideum | A(G)1-8 | Emery, H. S., & Weiner, A. M. (1981). An irregular satellite sequence is found at the termini of the linear extrachromosomal rDNA in Dictyostelium discoideum. Cell, 26(3 Pt 1), 411–419. | yes | / | / | / | / | / |
| **Plants** | Solanum lycopersicum (tomato) | TT[T/A]GGG | Ganal, M. W., Lapitan, N. L., & Tanksley, S. D. (1991). Macrostructure of the tomato telomeres. The Plant cell, 3(1), 87–94. | yes | TTTAGGG | yes | OU640346.1; OU640347.1; OU640349.1; OU640351.1; OU640353.1; OU640354.1; OU640344.1; OU640345.1; OU640352.1 | 9/12 | 48474 |
|  | Arabidopsis thaliana (thale cress) | TTTAGGG | Richards, E. J., & Ausubel, F. M. (1988). Isolation of a higher eukaryotic telomere from Arabidopsis thaliana. Cell, 53(1), 127–136. | yes | TTTAGGG | yes | CP087126.2;  CP087127.2;  CP087128.1;  CP087129.2;  CP087130.2 | 5/5 | 4480 |
| **Other Protists** | Plasmodium falciparum (human parasite) | TT[T/C]AGGG | Vernick, K. D., & McCutchan, T. F. (1988). Sequence and structure of a Plasmodium falciparum telomere. Molecular and biochemical parasitology, 28(2), 85–94. | yes | TT[T/C]AGGG | yes | NC_004325.2; NC_037280.1; NC_000521.4; NC_004318.2; NC_004326.2; NC_004327.3; NC_004328.3; NC_004329.3; NC_004330.2; NC_037281.1; NC_037282.1; NC_004331.3; NC_037283.1 | 13/15 | 4731 |
|  | Plasmodium berghei (rodent parasite) | TT[T/C]AGGG | Ponzi, M., Pace, T., Dore, E., & Frontali, C. (1985). Identification of a telomeric DNA sequence in Plasmodium berghei. The EMBO journal, 4(11), 2991–2995. | yes | TT[T/C]AGGG | yes | NC_036159.2; NC_036160.2; NC_036161.2; NC_036162.2; NC_036163.2; NC_036166.2; NC_036168.2; NC_036169.2 | 8/16 | 732 |
|  | Theileria annulata | TTTTAGGG | Sohanpal, B. K., Morzaria, S. P., Gobright, E. I., & Bishop, R. P. (1995). Characterisation of the telomeres at opposite ends of a 3 Mb Theileria parva chromosome. Nucleic acids research, 23(11), 1942–1947. | yes | TTTAGGG | no | NC_011100.1; NC_011098.1; NC_011129.2 | 3/4 | 1154 |
|  | Cryptosporidium parvum | TTTAGG | Liu, C., Schroeder, A. A., Kapur, V., & Abrahamsen, M. S. (1998). Telomeric sequences of Cryptosporidium parvum. Molecular and biochemical parasitology, 94(2), 291–296. | yes | TTTAGG | yes | NC_006981.1; NC_006982.1; NC_006983.1; NC_006984.1; NC_006985.1 | 5/8 | 2647 |
|  | Giardia lamblia | TTAGG | Morrison, H. G., McArthur, A. G., Gillin, F. D. (2007). Genomic minimalism in the early diverging intestinal parasite Giardia lamblia. Science (New York, N.Y.), 317(5846), 1921–1926. | yes | TAGGG | no | NC_051856.1; NC_051858.1; NC_051859.1 | 3/5 | 1772 |
|  | Giardia intestinalis | TAGGG | Le Blancq, S. M., Kase, R. S., & Van der Ploeg, L. H. (1991). Analysis of a Giardia lamblia rRNA encoding telomere with [TAGGG]n as the telomere repeat. Nucleic acids research, 19(20), 5790. | yes | TAGGG | yes | NC_051856.1; NC_051858.1; NC_051859.1 | 3/5 | 1772 |
|  | Leishmania major | TTAGGG | Teixeira, M.T., and Gilson, E. (2005). Telomere maintenance, function and evolution: the yeast paradigm. Chromosome Res 13, 535–548. 10.1007/s10577-005-0999-0. | yes | TTAGGG | yes | NC_004916.2; NC_007245.2; NC_007246.2; NC_007247.2; NC_007248.2; NC_007250.2; NC_007251.2; NC_007252.2; NC_007253.2; NC_007254.2; NC_007255.2; NC_007256.2; NC_007257.2; NC_007258.2; NC_007259.2; NC_007260.2; NC_007261.2; NC_007262.2; NC_007263.2; NC_007264.2; NC_007265.2; NC_007266.2; NC_007267.2; NC_007268.2; NC_007269.2; NC_007270.2; NC_007271.2; NC_007272.2; NC_007273.2; NC_007285.2; NC_007286.2; NC_007284.2; NC_007287.2 | 33/36 | 1321 |
|  | Trypanosoma brucei | TTAGGG | Blackburn, E. H., & Challoner, P. B. (1984). Identification of a telomeric DNA sequence in Trypanosoma brucei. Cell, 36(2), 447–457. | yes | TTAGGG | yes | NC_008409.1; NC_007283.1 | 2/10 | 482 |

Table S2: Detect telomeric motif sequence of fungi by TelFinder

| species | abbr. | motif | len | subphylum | IFdetection | chr with telomere | telchrN/allchrN | max Tellen |
| --- | --- | --- | --- | --- | --- | --- | --- | --- |
| Peltaster fructicola | PFA | TAGGG | 5 | Pezizomycotina | yes | CP051139.1;CP051140.1; CP051141.1;CP051142.1; CP051143.1 | 5/5 | 119 |
| Zymoseptoria tritici | ZTR | TTAGGG | 6 | Pezizomycotina | yes | CM009548.1;CM009544.1; CM009545.1;CM009546.1; CM009541.1;CM009552.1; CM009550.1;CM009539.1; CM009551.1;CM009554.1; CM009547.1;CM009542.1; CM009553.1;CM009556.1; CM009540.1;CM009549.1; CM009543.1;CM009555.1 | 18/18 | 270 |
| Cercospora sojina | CSO | / | 0 | Pezizomycotina | no | / | / | / |
| Cercospora beticola | CBT | / | 0 | Pezizomycotina | no | / | / | / |
| Venturia effusa | VEF | TTAGGG | 6 | Pezizomycotina | yes | CP042199.1;CP042187.1; CP042204.1;CP042192.1; CP042185.1;CP042188.1; CP042194.1;CP042190.1; CP042195.1;CP042196.1; CP042191.1;CP042189.1; CP042203.1;CP042193.1; CP042198.1;CP042200.1; CP042201.1;CP042202.1; CP042197.1;CP042186.1 | 20/21 | 168 |
| Pyrenophora teres | PTE | TTAGGG | 6 | Pezizomycotina | yes | CM017824.1;CM017827.1; CM017826.1;CM017821.1; CM017831.1;CM017825.1; CM017829.1;CM017822.1; CM017830.1;CM017820.1; CM017828.1;CM017823.1 | 12/12 | 144 |
| Bipolaris sorokiniana | BSC | TTAGGG | 6 | Pezizomycotina | yes | CM018003.1;CM018017.1 | 2/16 | 30 |
| Alternaria solani | ASL | TTAGGG | 6 | Pezizomycotina | yes | CP022024.1;CP022025.1; CP022026.1;CP022027.1; CP022028.1;CP022029.1; CP022030.1;CP022031.1; CP022032.1;CP022033.1 | 10/10 | 126 |
| Alternaria brassicae | ABR | TAAGGG | 6 | Pezizomycotina | yes | CM016504.1;CM016505.1; CM016506.1;CM016507.1; CM016508.1;CM016509.1; CM016510.1;CM016511.1; CM016512.1;CM016513.1 | 10/10 | 150 |
| Parastagonospora nodorum | PNO | TTAGGG | 6 | Pezizomycotina | yes | CP022852.1;CP022853.1; CP022855.1;CP022854.1; CP022856.1;CP022858.1; CP022857.1;CP022860.1; CP022859.1;CP022861.1; CP022863.1;CP022862.1; CP022865.1;CP022864.1; CP022868.1;CP022866.1; CP022870.1;CP022873.1; CP022871.1;CP022867.1 | 22/22 | 317 |
| Blumeria graminis | BGT | TTAGGG | 6 | Pezizomycotina | yes | LR026984.1;LR026986.1; LR026988.1;LR026989.1; LR026991.1;LR026992.1; LR026993.1 | 7/12 | 421 |
| Botrytis cinerea | BFU | TTAGGG | 6 | Pezizomycotina | yes | NC_037312.1;NC_037316.1; NC_037322.1;NC_037320.1; NC_037325.1;NC_037311.1; NC_037317.1;NC_037321.1; NC_037324.1;NC_037315.1; NC_037318.1;NC_037310.1; NC_037323.1;NC_037313.1; NC_037319.1;NC_037327.1; NC_037326.1 | 17/18 | 129 |
| Sclerotinia sclerotiorum | SSL | TTAGGG | 6 | Pezizomycotina | yes | CP017828.1;CP017815.1; CP017822.1;CP017818.1; CP017823.1;CP017829.1; CP017827.1;CP017825.1; CP017817.1;CP017821.1; CP017814.1;CP017819.1; CP017820.1;CP017816.1 | 14/16 | 195 |
| Pyricularia pennisetigena | PPE | TTAGGG | 6 | Pezizomycotina | yes | NC_043742.1;NC_043743.1; NC_043744.1 | 3/5 | 102 |
| Pyricularia oryzae | POR | TTAGGG | 6 | Pezizomycotina | yes | CP050920.1;CP050921.1; CP050922.1;CP050923.1; CP050924.1;CP050925.1; CP050926.1;CP050927.1; CP050928.1 | 9/9 | 196 |
| Pyricularia grisea | PGR | TTAGGG | 6 | Pezizomycotina | yes | NC_044975.1 | 1/3 | 114 |
| Valsa mali | VMA | TTAGGG | 6 | Pezizomycotina | yes | CM003098.1;CM003099.1; CM003105.1 | 3/13 | 150 |
| Colletotrichum higginsianum | COI | TTAGGG | 6 | Pezizomycotina | yes | NC_030954.1;NC_030955.1; NC_030956.1;NC_030957.1; NC_030958.1;NC_030959.1 | 6/6 | 138 |
| Verticillium dahliae | VDA | TTAGGG | 6 | Pezizomycotina | yes | CM014044.1 | 1/8 | 459 |
| Drechmeria coniospora | DCO | CCGTTGCTGTTG | 12 | Pezizomycotina | yes | CM004174.1;CM004175.1; CM004176.1 | 3/3 | 333 |
| Cordyceps militaris | CMT | TTAGGG | 6 | Pezizomycotina | yes | CP023328.1;CP023327.1; CP023326.1;CP023322.1; CP023325.1;CP023323.1; CP023324.1 | 7/7 | 156 |
| Trichoderma reesei | TRE | TTAGGG | 6 | Pezizomycotina | yes | CP021297.1;CP021298.1; CP021299.1;CP021300.1; CP021301.1;CP021302.1 | 6/7 | 94 |
| Metarhizium brunneum | MBR | TTAGGG | 6 | Pezizomycotina | yes | CP058932.1;CP058933.1; CP058934.1;CP058935.1; CP058936.1;CP058937.1; CP058938.1 | 7/8 | 150 |
| Pochonia chlamydosporia | PCH | TTAGGG | 6 | Pezizomycotina | yes | NC_035790.1;NC_035793.1; NC_035794.1;NC_035795.1; NC_035796.1 | 5/7 | 156 |
| Epichloe festucae | EFE | TTAGGG | 6 | Pezizomycotina | yes | CP031385.1;CP031386.1; CP031387.1;CP031388.1; CP031389.1;CP031390.1; CP031391.1 | 7/8 | 228 |
| Fusarium verticillioides | FVR | / | 0 | Pezizomycotina | no | / | / | / |
| Fusarium pseudograminearum | FPU | TTAGGG | 6 | Pezizomycotina | yes | NC_031951.1;NC_031952.1; NC_031954.1 | 3/4 | 102 |
| Fusarium venenatum | FVE | TTAGGG | 6 | Pezizomycotina | yes | NC_038012.1;NC_038013.1; NC_038014.1;NC_038015.1 | 4/5 | 156 |
| Fusarium circinatum | FCI | / | 0 | Pezizomycotina | no | / | / | / |
| Fusarium graminearum | FGR | TTAGGG | 6 | Pezizomycotina | yes | HG970332.2;HG970333.2; HG970334.2;HG970335.2 | 4/6 | 144 |
| Fusarium culmorum | FCU | TTAGGG | 6 | Pezizomycotina | yes | CP064747.1;CP064748.1; CP064749.1;CP064750.1 | 4/6 | 276 |
| Fusarium oxysporum | FOX | TTAGGG | 6 | Pezizomycotina | yes | NC_031000.1;NC_030990.1 | 2/15 | 168 |
| Thermothielavioides terrestris | TTT | TTAGGG | 6 | Pezizomycotina | yes | NC_016457.1;NC_016458.1; NC_016459.1;NC_016460.1; NC_016461.1;NC_016462.1 | 6/6 | 315 |
| Thermothelomyces thermophilus | TTH | TTAGGG | 6 | Pezizomycotina | yes | NC_016472.1;NC_016473.1; NC_016474.1;NC_016475.1; NC_016476.1;NC_016477.1; NC_016478.1 | 7/7 | 193 |
| Podospora comata | PCO | / | 0 | Pezizomycotina | no | / | / | / |
| Neurospora crassa | NCR | TTAGGG | 6 | Pezizomycotina | yes | NC_026501.1;NC_026502.1; NC_026503.1;NC_026504.1; NC_026505.1;NC_026506.1; NC_026507.1 | 7/8 | 312 |
| Exophiala lecanii-corni | ELC | TTAGGG | 6 | Pezizomycotina | yes | CP034370.1;CP034371.1; CP034372.1;CP034373.1; CP034374.1;CP034375.1; CP034377.1;CP034378.1; CP034379.1;CP034380.1; CP034381.1;CP034376.1 | 12/13 | 92 |
| Talaromyces pinophilus | TPI | TTAGGG | 6 | Pezizomycotina | yes | CP017344.1;CP017345.1; CP017346.1;CP017347.1; CP017348.1;CP017349.1; CP017350.1;CP017351.1 | 8/9 | 141 |
| Talaromyces rugulosus | TRU | TTAGGG | 6 | Pezizomycotina | yes | NC_049561.1;NC_049562.1; NC_049563.1;NC_049564.1; NC_049565.1;NC_049566.1 | 6/6 | 180 |
| Talaromyces marneffei | TMF | TTAGG[G,A] | 10 | Pezizomycotina | yes | CP015868.1;CP015869.1; CP015871.1;CP015872.1; CP015873.1;CP015875.1 | 6/8 | 800 |
| Talaromyces funiculosus | TFU | TTAGGG | 6 | Pezizomycotina | yes | CP036227.1;CP036228.1; CP036229.1;CP036231.1; CP036232.1 | 5/21 | 126 |
| Penicillium polonicum | PPO | TTTAGGG | 7 | Pezizomycotina | yes | CM024080.1;CM024081.1; CM024082.1 | 3/4 | 182 |
| Monascus purpureus | MPU | / | 0 | Pezizomycotina | no | / | / | / |
| Aspergillus fumigatus | AFM | TTAGGG | 6 | Pezizomycotina | yes | NC_007194.1;NC_007195.1; NC_007196.1;NC_007197.1; NC_007198.1;NC_007199.1; NC_007200.1;NC_007201.1 | 8/8 | 119 |
| Aspergillus nidulans | ANI | TTAGGG | 6 | Pezizomycotina | yes | BN001302.1;BN001305.1; BN001306.1;BN001307.1; BN001308.1 | 5/8 | 230 |
| Aspergillus sojae | ASO | TTAGGGTCAACA | 12 | Pezizomycotina | yes | CP035525.1;CP035526.1; CP035527.1;CP035530.1 | 4/8 | 150 |
| Aspergillus parasiticus | APA | TTAGGGTCAACA | 12 | Pezizomycotina | yes | CP051028.1;CP051029.1; CP051030.1;CP051032.1; CP051033.1;CP051034.1 | 6/8 | 85 |
| Aspergillus oryzae | AOR | TTAGGGTCAACA | 12 | Pezizomycotina | yes | NC_036435.1;NC_036438.1; NC_036439.1;NC_036441.1; NC_036442.1 | 5/9 | 89 |
| Aspergillus flavus | AFV | TTAGGGTCAACA | 12 | Pezizomycotina | yes | CP061804.1;CP061805.1; CP061806.1;CP061807.1; CP061809.1;CP061810.1 | 6/9 | 260 |
| Sugiyamaella lignohabitans | SLB | / | 0 | Saccharomycotina | no | / | / | / |
| [Candida] hispaniensis | CHI | TTGACGAGAG | 10 | Saccharomycotina | yes | LS992270.1 | 1/6 | 207 |
| [Candida] auris | CAU | TTAGGTGGTGTCTGGGTTTC | 20 | Saccharomycotina | yes | CP060360.1;CP060361.1; CP060362.1;CP060363.1; CP060364.1;CP060365.1; CP060366.1 | 6/7 | 397 |
| [Candida] intermedia | CIN | TTAGGGAGGTAGAGGTTTTTC | 21 | Saccharomycotina | yes | LT635756.1;LT635757.1; LT635758.1;LT635759.1; LT635760.1;LT635761.1; LT635762.1 | 7/8 | 400 |
| Clavispora lusitaniae | CLU | TTAGGGAGGTACTGATGTTCT | 21 | Saccharomycotina | yes | CP038484.1;CP038485.1; CP038486.1;CP038487.1; CP038489.1;CP038490.1; CP038491.1;CP038488.1 | 8/8 | 396 |
| Metschnikowia aff. pulcherrima | MAP | TTAGGGAGGTACGGGTGTCTTAGCATC | 27 | Saccharomycotina | yes | CP034456.1;CP034457.1; CP034458.1 | 3/7 | 343 |
| Metschnikowia reukaufii | MRE | TTAGGGATGTACTGATTTATC | 21 | Saccharomycotina | yes | CM010597.1;CM010598.1; CM010604.1 | 3/9 | 406 |
| Yarrowia lipolytica | YLI | TTAGTCAGGG | 10 | Saccharomycotina | yes | CP061012.1;CP061014.1; CP061015.1;CP061016.1; CP061017.1 | 5/7 | 663 |
| Saccharomycopsis malanga | SMA | TAAGGGTGTCAGTGGGG | 17 | Saccharomycotina | yes | CP025321.1;CP025322.1; CP025323.1;CP025324.1; CP025325.1;CP025326.1 | 6/7 | 227 |
| Saccharomycopsis fibuligera | SFI | TAAGGGTGGTG | 11 | Saccharomycotina | yes | CP015978.1;CP015983.1; CP015984.1 | 3/7 | 146 |
| Hyphopichia pseudoburtonii | HPS | TACGGGTCTTTTCTACGAGGGTGAGGAGG | 29 | Saccharomycotina | yes | CP024751.1;CP024752.1; CP024753.1;CP024754.1; CP024755.1;CP024756.1; CP024757.1;CP024758.1 | 8/8 | 816 |
| Hyphopichia burtonii | HBU | / | 0 | Saccharomycotina | no | / | / | / |
| Candida orthopsilosis | COT | / | 0 | Saccharomycotina | no | / | / | / |
| Candida dubliniensis | CDU | TACGGATGTCTAACTTCTGGTG | 22 | Saccharomycotina | yes | NC_012867.1 | 1/8 | 254 |
| Candida tropicalis | CTP | TAAGGATGTCACGATCATTGGTG | 23 | Saccharomycotina | yes | CP047869.1;CP047870.1; CP047871.1;CP047872.1; CP047873.1;CP047874.1; CP047875.1 | 7/7 | 199 |
| Candida albicans | CAL | TACGGATGTCTAACTTCTTGGTG | 23 | Saccharomycotina | yes | CP032017.1 | 1/8 | 931 |
| Debaryomyces hansenii | DHA | TAGGGATGTTGAGGTG | 16 | Saccharomycotina | yes | NC_006044.2;NC_006048.2 | 2/8 | 315 |
| Scheffersomyces stipitis | SST | TATGGATCTTTTCACGTCTTGCGG | 24 | Saccharomycotina | yes | NC_009068.1;NC_009042.1; NC_009043.1;NC_009044.1; NC_009045.1;NC_009046.1; NC_009047.1;NC_009048.1 | 8/8 | 558 |
| Millerozyma farinosa | MFA | / | 0 | Saccharomycotina | no | / | / | / |
| Saccharomycetaceae sp. | AAC | TATGGGTCTCTCAGCGGTGTGGTG | 24 | Saccharomycotina | yes | CP006020.1;CP006021.1; CP006022.1;CP006023.1; CP006025.1;CP006026.1 | 6/8 | 697 |
| Kazachstania naganishii | KNA | / | 0 | Saccharomycotina | no | / | / | / |
| Kazachstania africana | KAF | / | 0 | Saccharomycotina | no | / | / | / |
| Tetrapisispora blattae | TBL | / | 0 | Saccharomycotina | no | / | / | / |
| Tetrapisispora phaffii | TPF | / | 0 | Saccharomycotina | no | / | / | / |
| Zygotorulaspora mrakii | ZMR | TAGGGGTGCGGTG | 13 | Saccharomycotina | yes | NC_050719.1;NC_050720.1; NC_050722.1;NC_050723.1; NC_050724.1;NC_050726.1 | 6/8 | 355 |
| Eremothecium cymbalariae | ERC | TACGGGTCTCTCAGCGGTGTGGTG | 24 | Saccharomycotina | yes | NC_016451.1;NC_016452.1; NC_016453.1;NC_016454.1; NC_016456.1 | 5/8 | 298 |
| Eremothecium gossypii | AGO | TACGGGTCTCTCAGCGGTGTGGTG | 24 | Saccharomycotina | yes | NC_005782.2;NC_005783.5; NC_005784.3;NC_005785.6; NC_005786.2;NC_005787.5; NC_005788.4 | 7/8 | 672 |
| Naumovozyma dairenensis | NDI | / | 0 | Saccharomycotina | no | / | / | / |
| Naumovozyma castellii | NCS | / | 0 | Saccharomycotina | no | / | / | / |
| [Candida] glabrata | CGR | TCTGGGTGCTGTGGGG | 16 | Saccharomycotina | yes | CP048234.1;CP048230.1; CP048240.1;CP048241.1; CP048236.1;CP048235.1; CP048232.1;CP048238.1; CP048233.1;CP048237.1; CP048231.1;CP048239.1; CP048242.1 | 13/13 | 817 |
| Zygosaccharomyces parabailii | ZPA | / | 0 | Saccharomycotina | no | / | / | / |
| Zygosaccharomyces rouxii | ZRO | / | 0 | Saccharomycotina | no | / | / | / |
| Torulaspora delbrueckii | TDL | TAAGGTTGTGGTG | 13 | Saccharomycotina | yes | NC_016501.1 | 1/8 | 214 |
| Lachancea mirantina | LMI | TGGAGGAGGAGTG | 13 | Saccharomycotina | yes | LT598466.1;LT598469.1 | 2/8 | 52 |
| Lachancea dasiensis | LDA | / | 0 | Saccharomycotina | no | / | / | / |
| Lachancea nothofagi | LNO | / | 0 | Saccharomycotina | no | / | / | / |
| Lachancea thermotolerans | LTH | GTGGAGTAC | 9 | Saccharomycotina | yes | NC_013077.1;NC_013082.1; NC_013084.1 | 3/8 | 153 |
| Lachancea meyersii | LME | / | 0 | Saccharomycotina | no | / | / | / |
| Lachancea fermentati | LFE | / | 0 | Saccharomycotina | no | / | / | / |
| Lachancea kluyveri | LKL | TGGACATGCGTACTGTGAGGTCTGGG | 26 | Saccharomycotina | yes | CM000687.1;CM000688.1; CM000689.1;CM000691.1 | 4/8 | 648 |
| Saccharomyces eubayanus | SEU | T(G)2-3(TG)1-6 | 5~16 | Saccharomycotina | / | / | / | / |
| Saccharomyces arboricola | SAR | T(G)2-3(TG)1-6 | 5~16 | Saccharomycotina | / | / | / | / |
| Saccharomyces boulardii | SBO | T(G)2-3(TG)1-6 | 5~16 | Saccharomycotina | / | / | / | / |
| Saccharomyces kudriavzevii | SKU | T(G)2-3(TG)1-6 | 5~16 | Saccharomycotina | / | / | / | / |
| Saccharomyces pastorianus | SPS | T(G)2-3(TG)1-6 | 5~16 | Saccharomycotina | / | / | / | / |
| Saccharomyces paradoxus | SPR | T(G)2-3(TG)1-6 | 5~16 | Saccharomycotina | / | / | / | / |
| Saccharomyces cerevisiae | SCE | T(G)2-3(TG)1-6 | 5~16 | Saccharomycotina | / | / | / | / |
| Kluyveromyces lactis | KLA | TTAGGTATGTGGTGTACGGATTTGA | 25 | Saccharomycotina | yes | NC_006037.1;NC_006038.1; NC_006039.1;NC_006040.1; NC_006041.1;NC_006042.1 | 6/8 | 708 |
| Kluyveromyces marxianus | KMX | TTAGGTATGTGGTGTACGGATTTGA | 25 | Saccharomycotina | yes | NC_036030.1;NC_036025.1; NC_036028.1;NC_036029.1; NC_036032.1;NC_036027.1; NC_036031.1;NC_036026.1 | 8/9 | 750 |
| Ogataea parapolymorpha | OPA | GGTGGCGG | 8 | Saccharomycotina | yes | NC_027860.1;NC_027863.1 | 2/7 | 78 |
| Pichia kudriavzevii | PKZ | GTGTGTTACAATATGAACTAGGAGCGAG | 28 | Saccharomycotina | yes | NC_042507.1;NC_042509.1; NC_042510.1 | 3/5 | 529 |
| Komagataella phaffii | PPA | ATGCTGG | 7 | Saccharomycotina | yes | LT962476.1;LT962477.1; LT962478.1;LT962479.1 | 4/4 | 237 |
| Komagataella pastoris | KPA | ATGCTGG | 7 | Saccharomycotina | yes | CP014584.1;CP014585.1; CP014587.1;CP014588.1; CP014593.1;CP014586.1 | 6/11 | 357 |
| Cyberlindnera jadinii | CJA | TCTGGGTG | 8 | Saccharomycotina | yes | DG000065.1;DG000066.1; DG000067.1;DG000069.1; DG000070.1 | 5/13 | 443 |
| Schizosaccharomyces pombe | SPO | G2–8TTAC(A) | 0 | Taphrinomycotina | no | / | / | / |
| Pyricularia sp. | PYR | TTAGGG | 6 | Pezizomycotina | yes | RRCJ01000018.1;  RRCJ01000016.1; RRCJ01000013.1  ;RRCJ01000043.1; RRCJ01000044.1;  RRCJ01000047.1; RRCJ01000004.1;  RRCJ01000007.1; RRCJ01000011.1;  RRCJ01000015.1; RRCJ01000020.1;  RRCJ01000025.1; RRCJ01000031.1 | 13/49 | 102 |
| Byssochlamys spectabilis | BSP | TTTAGGG | 7 | Pezizomycotina | yes | CM012214.1;CM012215.1; CM012216.1;CM012217.1; CM012218.1;CM012219.1; CM012220.1;CM012221.1 | 8/10 | 112 |
| Exserohilum turcica | ETE | TTAGGG | 6 | Pezizomycotina | yes | CP054649.1;CP054643.1; CP054652.1;CP054650.1; CP054640.1;CP054627.1; CP054628.1;CP054630.1; CP054636.1;CP054654.1; CP054635.1;CP054638.1; CP054633.1;CP054642.1; CP054653.1;CP054641.1; CP054647.1;CP054639.1; CP054632.1;CP054644.1; CP054629.1;CP054637.1; CP054646.1;CP054634.1 | 24/30 | 181 |
| Antonospora locustae | ALO | / | 0 | Fungi incertae sedis | no | / | / | / |
| Encephalitozoon romaleae | ERO | / | 0 | Fungi incertae sedis | no | / | / | / |
| Encephalitozoon intestinalis | EIN | / | 0 | Fungi incertae sedis | no | / | / | / |
| Encephalitozoon hellem | EHE | / | 0 | Fungi incertae sedis | no | / | / | / |
| Encephalitozoon cuniculi | ECU | GGTTATGTGT | 10 | Fungi incertae sedis | yes | NC_003242.2 | 1/11 | 70 |
| Pleurotus ostreatus | POS | TTAGGG | 6 | Agaricomycotina;Basidiomycota | yes | CM016808.1;CM016811.1; CM016812.1;CM016813.1; CM016814.1;CM016816.1; CM016817.1 | 7/11 | 48 |
| Pyrrhoderma noxium | PNX | TGTTAGG | 7 | Agaricomycotina;Basidiomycota | yes | CM008251.1;CM008252.1; CM008254.1;CM008255.1; CM008256.1;CM008258.1; CM008260.1 | 7/13 | 463 |
| Ustilago maydis | UMA | TTAGGG | 6 | Ustilaginomycotina;Basidiomycota | yes | NC_026481.1;NC_026497.1 | 2/23 | 372 |
| Ustilago bromivora | UBR | TTAGGG | 6 | Ustilaginomycotina;Basidiomycota | yes | LT558129.1;LT558126.1; LT558118.1;LT558125.1; LT558134.1;LT558120.1; LT558121.1;LT558127.1; LT558137.1;LT558122.1; LT558135.1;LT558128.1; LT558138.1;LT558124.1; LT558133.1;LT558136.1; LT558130.1;LT558123.1; LT558119.1;LT558117.1; LT558139.1;LT558131.1 | 22/24 | 208 |
| Trametes hirsuta | THI | TTAGGG | 6 | Agaricomycotina;Basidiomycota | yes | CP019373.1;CP019382.1; CP019376.1;CP019377.1; CP019381.1;CP019378.1; CP019371.1;CP019372.1; CP019380.1;CP019379.1; CP019375.1;CP019370.1; CP019374.1 | 13/13 | 286 |
| Sporisorium reilianum | SRE | / | 0 | Ustilaginomycotina;Basidiomycota | no | / | / | / |
| Sporisorium scitamineum | SSC | TTAGGG | 6 | Ustilaginomycotina;Basidiomycota | yes | CP010923.1;CP010916.1; CP010927.1;CP010913.1; CP010919.1;CP010928.1; CP010917.1;CP010925.1; CP010924.1;CP010915.1; CP010934.1;CP010935.1; CP010914.1;CP010931.1; CP010926.1;CP010930.1; CP010929.1;CP010938.1; CP010932.1;CP010936.1; CP010918.1;CP010922.1; CP010921.1;CP010920.1 | 24/27 | 139 |
| Sporisorium graminicola | SGR | TTAGGG | 6 | Ustilaginomycotina;Basidiomycota | yes | NC_043737.1;NC_043735.1; NC_043726.1;NC_043738.1; NC_043727.1;NC_043732.1; NC_043719.1;NC_043721.1; NC_043736.1;NC_043734.1; NC_043723.1;NC_043722.1; NC_043731.1;NC_043730.1; NC_043725.1;NC_043729.1; NC_043728.1;NC_043733.1; NC_043737.1;NC_043720.1; NC_043724.1;NC_043739.1 | 22/22 | 328 |
| Malassezia restricta | MRT | TTAGTG | 6 | Ustilaginomycotina;Basidiomycota | yes | NC_040194.1;NC_040193.1; NC_040195.1;NC_040194.1; NC_040198.1;NC_040196.1; NC_040200.1;NC_040199.1; NC_040201.1;NC_040197.1 | 10/10 | 312 |
| Malassezia globosa | MGL | TTAGTG | 6 | Ustilaginomycotina;Basidiomycota | yes | CP046436.1;CP046435.1; CP046439.1;CP046433.1; CP046431.1;CP046432.1; CP046437.1;CP046436.1; CP046434.1;CP046438.1; CP046440.1 | 10/11 | 209 |
| Malassezia sympodialis | MSYM | TTAAGTG | 7 | Ustilaginomycotina;Basidiomycota | yes | LT671791.1;LT671792.1; LT671795.1;LT671794.1; LT671789.1;LT671790.1; LT671793.1;LT671796.1 | 8/8 | 285 |
| Malassezia furfur | MFU | TTAGGA | 6 | Ustilaginomycotina;Basidiomycota | yes | CP046237.1;CP046234.1; CP046239.1;CP046236.1; CP046238.1;CP046235.1; CP046240.1;CP046237.1 | 8/8 | 431 |
| Hericium erinaceus | HER | TTGGA | 5 | Agaricomycotina;Basidiomycota | yes | CM021160.1;CM021163.1; CM021166.1;CM021167.1; CM021169.1;CM021170.1; CM021172.1;CM021173.1 | 8/15 | 65 |
| Flammulina velutipes | FVL | TTAGGG | 6 | Agaricomycotina;Basidiomycota | yes | CM022370.1;CM022377.1; CM022376.1;CM022373.1; CM022375.1;CM022372.1; CM022374.1;CM022378.1; CM022371.1;CM022369.1; CM022370.1 | 11/11 | 286 |
| Cryptococcus wingfieldii | CWI | TTAGGGGG | 8 | Agaricomycotina;Basidiomycota | no | / | / | / |
| Cryptococcus neoformans | CNV | TTAGGGGG | 8 | Agaricomycotina;Basidiomycota | yes | NC_006679.1;NC_006684.1; NC_006685.1;NC_006694.1; NC_006693.1;NC_006680.1; NC_006692.1;NC_006686.1; NC_006687.1;NC_006670.1; NC_006683.1;NC_006681.1; NC_006682.1;NC_006691.1 | 14/14 | 96 |
| Cryptococcus gattii | CGI | TTAGGGGG | 8 | Agaricomycotina;Basidiomycota | yes | NC_014939.1;NC_014947.1; NC_014949.1;NC_014940.1; NC_014946.1;NC_014950.1; NC_014948.1;NC_014944.1; NC_014951.1;NC_014938.1; NC_014942.1 | 11/14 | 104 |
| Cryptococcus floricola | CFL | TTAGCGGGG | 9 | Agaricomycotina;Basidiomycota | yes | CM016930.1;CM016932.1; CM016940.1;CM016939.1; CM016942.1;CM016934.1; CM016931.1;CM016933.1; CM016938.1;CM016936.1 | 10/14 | 91 |
| Cryptococcus cf. gattii | CGA | TTAG(3-4) | 7 | Agaricomycotina;Basidiomycota | yes | CM018872.1;CM018860.1; CM018868.1;CM018865.1; CM018864.1;CM018861.1; CM018867.1;CM018870.1; CM018866.1;CM018869.1; CM018871.1;CM018862.1; CM018863.1 | 13/13 | 79 |
| Apiotrichum mycotoxinovorans | AMY | TTAGGGG | 7 | Agaricomycotina;Basidiomycota | yes | CP053620.1;CP053624.1 | 2/7 | 97 |
| Agaricus bisporus | ABP | TTAGGGGG | 8 | Agaricomycotina;Basidiomycota | yes | CP015481.1;CP015476.1; CP015482.1;CP015479.1; CP015472.1;CP015471.1; CP015470.1;CP015473.1; CP015474.1;CP015477.1 | 10/13 | 88 |
| Hypsizygus marmoreus | HMA | TTAGGG | 6 | Agaricomycotina;Basidiomycota | yes | CM024087.1;CM024086.1; CM024085.1;CM024093.1; CM024095.1;CM024090.1; CM024092.1;CM024091.1; CM024088.1;CM024094.1; CM024089.1;CM024084.1 | 12/12 | 145 |

Table S3A: SV frequency of telomere (HSA)

| end | chr_name | chr | telomere length | motif count | SV count | SV frequency |
| --- | --- | --- | --- | --- | --- | --- |
| left | NC_060925.1 | chr1 | 2700 | 438 | 98 | 0.22 |
| left | NC_060926.1 | chr2 | 3616 | 570 | 175 | 0.31 |
| left | NC_060927.1 | chr3 | 2636 | 436 | 70 | 0.16 |
| left | NC_060928.1 | chr4 | 3258 | 533 | 120 | 0.23 |
| left | NC_060929.1 | chr5 | 2295 | 367 | 53 | 0.14 |
| left | NC_060930.1 | chr6 | 2892 | 475 | 36 | 0.08 |
| left | NC_060931.1 | chr7 | 3412 | 554 | 93 | 0.17 |
| left | NC_060932.1 | chr8 | 2512 | 405 | 104 | 0.26 |
| left | NC_060933.1 | chr9 | 3630 | 585 | 119 | 0.20 |
| left | NC_060934.1 | chr10 | 2636 | 429 | 68 | 0.16 |
| left | NC_060935.1 | chr11 | 1983 | 319 | 35 | 0.11 |
| left | NC_060936.1 | chr12 | 3101 | 487 | 124 | 0.25 |
| left | NC_060937.1 | chr13 | 2541 | 423 | 7 | 0.02 |
| left | NC_060938.1 | chr14 | 2073 | 344 | 4 | 0.01 |
| left | NC_060939.1 | chr15 | 3253 | 531 | 161 | 0.30 |
| left | NC_060940.1 | chr16 | 2268 | 374 | 71 | 0.19 |
| left | NC_060941.1 | chr17 | 2205 | 364 | 76 | 0.21 |
| left | NC_060942.1 | chr18 | 2942 | 336 | 20 | 0.06 |
| left | NC_060943.1 | chr19 | 2281 | 357 | 68 | 0.19 |
| left | NC_060944.1 | chr20 | 2723 | 443 | 66 | 0.15 |
| left | NC_060945.1 | chr21 | 3008 | 460 | 53 | 0.12 |
| left | NC_060946.1 | chr22 | 4574 | 736 | 74 | 0.10 |
| left | NC_060947.1 | chrX | 1822 | 302 | 37 | 0.12 |
| left | NC_060948.1 | chrY | 5654 | 941 | 34 | 0.04 |
| right | NC_060925.1 | chr1 | 3185 | 512 | 102 | 0.20 |
| right | NC_060926.1 | chr2 | 2621 | 424 | 64 | 0.15 |
| right | NC_060927.1 | chr3 | 4612 | 746 | 172 | 0.23 |
| right | NC_060928.1 | chr4 | 2296 | 373 | 45 | 0.12 |
| right | NC_060929.1 | chr5 | 1553 | 255 | 42 | 0.16 |
| right | NC_060930.1 | chr6 | 2778 | 450 | 52 | 0.12 |
| right | NC_060931.1 | chr7 | 2219 | 357 | 70 | 0.20 |
| right | NC_060932.1 | chr8 | 1842 | 454 | 152 | 0.33 |
| right | NC_060933.1 | chr9 | 2970 | 474 | 153 | 0.32 |
| right | NC_060934.1 | chr10 | 3196 | 515 | 109 | 0.21 |
| right | NC_060935.1 | chr11 | 2589 | 422 | 44 | 0.10 |
| right | NC_060936.1 | chr12 | 2337 | 375 | 40 | 0.11 |
| right | NC_060937.1 | chr13 | 3499 | 572 | 61 | 0.11 |
| right | NC_060938.1 | chr14 | 1652 | 267 | 34 | 0.13 |
| right | NC_060939.1 | chr15 | 2929 | 464 | 61 | 0.13 |
| right | NC_060940.1 | chr16 | 2678 | 435 | 49 | 0.11 |
| right | NC_060941.1 | chr17 | 2996 | 476 | 52 | 0.11 |
| right | NC_060942.1 | chr18 | 3489 | 557 | 132 | 0.24 |
| right | NC_060943.1 | chr19 | 2934 | 465 | 57 | 0.12 |
| right | NC_060944.1 | chr20 | 3153 | 490 | 118 | 0.24 |
| right | NC_060945.1 | chr21 | 4548 | 730 | 79 | 0.11 |
| right | NC_060946.1 | chr22 | 2931 | 467 | 87 | 0.19 |
| right | NC_060947.1 | chrX | 2943 | 468 | 101 | 0.22 |
| right | NC_060948.1 | chrY | 6389 | 1023 | 113 | 0.11 |

Table S3B: SV frequency of telomere (ATH)

| end | chr_name | chr | telomere length | motif count | SV count | SV frequency |
| --- | --- | --- | --- | --- | --- | --- |
| left | CP087126.1 | chr1 | 3257 | 439 | 9 | 0.02 |
| left | CP087127.1 | chr2 | 2946 | 379 | 4 | 0.01 |
| left | CP087128.1 | chr3 | 3263 | 443 | 35 | 0.08 |
| left | CP087129.1 | chr4 | 0 | 0 | 0 | NAN |
| left | CP087130.1 | chr5 | 3274 | 460 | 12 | 0.03 |
| right | CP087126.1 | chr1 | 3562 | 508 | 2 | 0.00 |
| right | CP087127.1 | chr2 | 3611 | 497 | 84 | 0.17 |
| right | CP087128.1 | chr3 | 3006 | 424 | 11 | 0.03 |
| right | CP087129.1 | chr4 | 3385 | 467 | 64 | 0.14 |
| right | CP087130.1 | chr5 | 4480 | 475 | 30 | 0.06 |

Table S3C: SV frequency of telomere (YLI)

| end | chr_name | chr | telomere length | motif count | SV count | SV frequency |
| --- | --- | --- | --- | --- | --- | --- |
| left | CP061012.1 | chrA | 369 | 37 | 0 | 0.00 |
| left | CP061013.1 | chrB | 0 | 0 | 0 | NAN |
| left | CP061014.1 | chrC | 663 | 63 | 13 | 0.21 |
| left | CP061015.1 | chrD | 256 | 21 | 4 | 0.19 |
| left | CP061016.1 | chrE | 0 | 0 | 0 | NAN |
| left | CP061017.1 | chrF | 0 | 0 | 0 | NAN |
| right | CP061012.1 | chrA | 520 | 48 | 0 | 0.00 |
| right | CP061013.1 | chrB | 0 | 0 | 0 | NAN |
| right | CP061014.1 | chrC | 478 | 38 | 13 | 0.34 |
| right | CP061015.1 | chrD | 434 | 42 | 6 | 0.14 |
| right | CP061016.1 | chrE | 303 | 30 | 1 | 0.03 |
| right | CP061017.1 | chrF | 383 | 38 | 1 | 0.03 |

Table S4A: coefficient for kmer length and repeat times in scoring and its detection result in five end of ATH

| kmer length | repeat times | kmer | suppChr |
| --- | --- | --- | --- |
| 1 | 1 | CCCTAAA | 4 |
| 0.1 | 1 | CCCTAAA | 4 |
| 0.2 | 1 | CCCTAAA | 4 |
| 0.3 | 1 | CCCTAAA | 4 |
| 0.4 | 1 | CCCTAAA | 4 |
| 0.5 | 1 | CCCTAAA | 4 |
| 0.6 | 1 | CCCTAAA | 4 |
| 0.7 | 1 | CCCTAAA | 4 |
| 0.8 | 1 | CCCTAAA | 4 |
| 0.9 | 1 | CCCTAAA | 4 |
| 1 | 0.1 | CCCTAAA | 4 |
| 1 | 0.2 | CCCTAAA | 4 |
| 1 | 0.3 | CCCTAAA | 4 |
| 1 | 0.4 | CCCTAAA | 4 |
| 1 | 0.5 | CCCTAAA | 4 |
| 1 | 0.6 | CCCTAAA | 4 |
| 1 | 0.7 | CCCTAAA | 4 |
| 1 | 0.8 | CCCTAAA | 4 |
| 1 | 0.9 | CCCTAAA | 4 |
| 0.1 | 0.9 | CCCTAAA | 4 |
| 0.2 | 0.8 | CCCTAAA | 4 |
| 0.3 | 0.7 | CCCTAAA | 4 |
| 0.4 | 0.6 | CCCTAAA | 4 |
| 0.5 | 0.5 | CCCTAAA | 4 |
| 0.6 | 0.4 | CCCTAAA | 4 |
| 0.7 | 0.3 | CCCTAAA | 4 |
| 0.8 | 0.2 | CCCTAAA | 4 |
| 0.9 | 0.1 | CCCTAAA | 4 |

Table S4B: coefficient for kmer length and repeat times in scoring and its detection result in five end of YLI

| kmer length | repeat times | kmer | suppChr |
| --- | --- | --- | --- |
| 1 | 1 | ACTAACCCTG | 3 |
| 0.1 | 1 | ACTAACCCTG | 3 |
| 0.2 | 1 | ACTAACCCTG | 3 |
| 0.3 | 1 | ACTAACCCTG | 3 |
| 0.4 | 1 | ACTAACCCTG | 3 |
| 0.5 | 1 | ACTAACCCTG | 3 |
| 0.6 | 1 | ACTAACCCTG | 3 |
| 0.7 | 1 | ACTAACCCTG | 3 |
| 0.8 | 1 | ACTAACCCTG | 3 |
| 0.9 | 1 | ACTAACCCTG | 3 |
| 1 | 0.1 | ACTAACCCTG | 1 |
| 1 | 0.2 | ACTAACCCTG | 2 |
| 1 | 0.3 | ACTAACCCTG | 2 |
| 1 | 0.4 | ACTAACCCTG | 3 |
| 1 | 0.5 | ACTAACCCTG | 3 |
| 1 | 0.6 | ACTAACCCTG | 3 |
| 1 | 0.7 | ACTAACCCTG | 3 |
| 1 | 0.8 | ACTAACCCTG | 3 |
| 1 | 0.9 | ACTAACCCTG | 3 |
| 0.1 | 0.9 | ACTAACCCTG | 3 |
| 0.2 | 0.8 | ACTAACCCTG | 3 |
| 0.3 | 0.7 | ACTAACCCTG | 3 |
| 0.4 | 0.6 | ACTAACCCTG | 3 |
| 0.5 | 0.5 | ACTAACCCTG | 3 |
| 0.6 | 0.4 | ACTAACCCTG | 3 |
| 0.7 | 0.3 | ACTAACCCTG | 2 |
| 0.8 | 0.2 | ACTAACCCTG | 2 |
| 0.9 | 0.1 | ACTAACCCTG | 1 |

Table S5: the possible reason for detection failure by TelFinder

| count | **species** | **reason** |
| --- | --- | --- |
| 8 | *Saccharomyces eubayanus, Saccharomyces arboricola,Saccharomyces boulardii, Saccharomyces kudriavzevii, Saccharomyces pastorianus, Saccharomyces paradoxus, Saccharomyces paradoxus, Schizosaccharomyces pombe* | Variable telomeric  repeat sequence |
| 27 | *Cercospora sojina, Cercospora beticola, Fusarium verticillioides, Fusarium circinatum, Podospora comata, Podospora comata, Podospora comata, Hyphopichia burtonii, Candida orthopsilosis, Millerozyma farinosa, Kazachstania naganishii, Kazachstania africana, Tetrapisispora blattae, Tetrapisispora phaffii, Naumovozyma dairenensis, Naumovozyma castellii, Zygosaccharomyces parabailii, Zygosaccharomyces rouxii, Lachancea dasiensis, Lachancea nothofagi, Lachancea meyersii, Lachancea fermentati, Lachancea fermentati, Encephalitozoon romaleae, Encephalitozoon intestinalis, Encephalitozoon intestinalis, Sporisorium reilianum* | Too few repeat times to  distinguish |
